# DNA storage in thermoresponsive microcapsules for repeated random multiplexed data access

**DOI:** 10.1038/s41565-023-01377-4

**Published:** 2023-05-04

**Authors:** Bas W. A. Bögels, Bichlien H. Nguyen, David Ward, Levena Gascoigne, David P. Schrijver, Anna-Maria Makri Pistikou, Alex Joesaar, Shuo Yang, Ilja K. Voets, Willem J. M. Mulder, Andrew Phillips, Stephen Mann, Georg Seelig, Karin Strauss, Yuan-Jyue Chen, Tom F. A. de Greef

**Affiliations:** 1 Laboratory of Chemical Biology, Department of Biomedical Engineering, Eindhoven University of Technology, Eindhoven, The Netherlands; 2 Institute for Complex Molecular Systems (ICMS), Eindhoven University of Technology, Eindhoven, The Netherlands; 3 Computational Biology Group, Department of Biomedical Engineering, Eindhoven University of Technology, Eindhoven, The Netherlands; 4 Microsoft, Redmond, WA USA; 5 Paul G. Allen School of Computer Science and Engineering, University of Washington, Seattle, WA USA; 6 Laboratory of Self-Organizing Soft Matter, Department of Chemical Engineering and Chemistry, Eindhoven University of Technology, Eindhoven, The Netherlands; 7 Department of Internal Medicine and Radboud Center for Infectious Diseases (RCI), Radboud University Nijmegen Medical Centre, Nijmegen, The Netherlands; 8 Microsoft Research, Cambridge, UK; 9 Centre for Protolife Research and Centre for Organized Matter Chemistry, School of Chemistry, University of Bristol, Bristol, UK; 10 School of Materials Science and Engineering, Shanghai Jiao Tong University, Shanghai, People’s Republic of China; 11 Zhangjiang Institute for Advanced Study (ZIAS), Shanghai Jiao Tong University, Shanghai, People’s Republic of China; 12 Department of Electrical Engineering, University of Washington, Seattle, WA USA; 13 Institute for Molecules and Materials, Radboud University, Nijmegen, The Netherlands; 14 Center for Living Technologies, Eindhoven-Wageningen-Utrecht Alliance, Utrecht, The Netherlands

**Keywords:** Nanoparticles, Biomedical engineering, Mechanical properties, Bioconjugate chemistry

## Abstract

DNA has emerged as an attractive medium for archival data storage due to its durability and high information density. Scalable parallel random access to information is a desirable property of any storage system. For DNA-based storage systems, however, this still needs to be robustly established. Here we report on a thermoconfined polymerase chain reaction, which enables multiplexed, repeated random access to compartmentalized DNA files. The strategy is based on localizing biotin-functionalized oligonucleotides inside thermoresponsive, semipermeable microcapsules. At low temperatures, microcapsules are permeable to enzymes, primers and amplified products, whereas at high temperatures, membrane collapse prevents molecular crosstalk during amplification. Our data show that the platform outperforms non-compartmentalized DNA storage compared with repeated random access and reduces amplification bias tenfold during multiplex polymerase chain reaction. Using fluorescent sorting, we also demonstrate sample pooling and data retrieval by microcapsule barcoding. Therefore, the thermoresponsive microcapsule technology offers a scalable, sequence-agnostic approach for repeated random access to archival DNA files.

## Abstract (web summary)

Microcompartments with a temperature-responsive membrane are used to stably localize DNA-encoded files, which enables parallel, repeated polymerase-chain-reaction-based random access and DNA file sorting using fluorescent barcodes.

## Main

Even as the world generates increasingly more data, our capacity to store this information lags behind^1^. Because traditional long-term storage media such as hard discs or magnetic tape have limited durability and storage density, there is growing interest in small organic molecules^2,3^, polymers^4,5^ and, more recently, DNA^6–8^ as molecular data carriers. Its intrinsic capacity for information storage, longevity and high information density make DNA a prime candidate for archival digital data storage^9^. Notable progress has been made in coding schemes^7,10,11^ used to convert binary data to DNA, and the current best method can achieve a density of 17 exabytes per gram (ref. ^12^), which exceeds magnetic and optical alternatives by approximately six orders of magnitude^9^. Additionally, the long-term storage of DNA in natural fossils^13^ or synthetic protective shells^14–17^ enables the storage of data for larger timescales^16,18^ than typical magnetic data carriers^19^.

With the introduction of parallelized chemical^20,21^ and enzymatic^22–24^ synthesis, the large-scale production of DNA for data storage has become viable. Simultaneously, the next-generation sequencing of DNA using Illumina^25^ or nanopore^26^ sequencing has enabled the high-throughput reading of DNA sequences. By circumventing the need to sequence entire datasets encoded on DNA, polymerase chain reaction (PCR)-based random access can selectively retrieve encoded data from a complex pool of DNA files^8^. However, PCR has two disadvantages. The first is that a small fraction of the pool is irretrievably consumed during the amplification of data and the repeated copying of DNA skews sequence representation^7,27^. Second, the multiplexed retrieval of DNA-encoded files using PCR is hindered by PCR bias^28,29^ and artifact formation due to molecular crosstalk^30^. PCR bias originates from differences in length, sequence composition, guanine–cytosine content and secondary structure of DNA^29^. Although it is possible to mitigate the effects of artifact formation and PCR bias by a careful sequence design^31^ and the inclusion of additional physical and logical redundancies, this leads to an increased cost of DNA synthesis and sequencing^7,12,27^. Thus, current strategies for retrieving multiple DNA files are based on physically separating reactions and amplifying each file individually using multiple singleplex PCR reactions^8,32,33^. Parallel amplification in a single reaction vessel can be achieved using emulsion PCR, which segregates DNA templates using water-in-oil droplets and prevents artifact formation^34,35^. Although emulsion PCR has been employed in DNA data retrieval^8,31^, the elaborate workflow, non-reusable nature and large quantities of organic solvents required to form emulsions for each reaction make it unattractive for large-scale adoption.

**Fig. 1:**
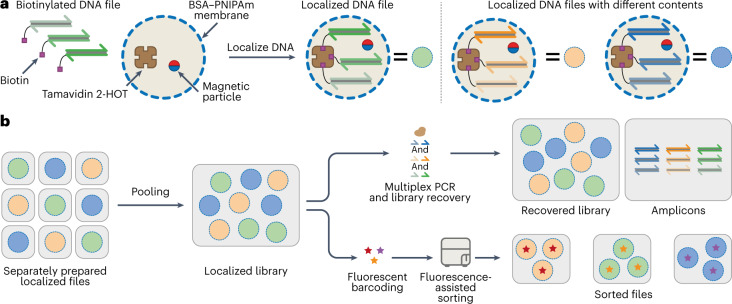
Proteinosomes as a platform for DNA data storage. **a**, Schematic of proteinosomes used for DNA-encoded data storage. Proteinosomes with BSA–PNIPAm-based thermoresponsive membranes^36^ encapsulate Tamavidin 2-HOT^44^ and magnetic particles (Methods). Digital data can be encoded into multiple fixed-length DNA sequences that are appended with forward and reverse primer sites to create a DNA-encoded file (Supplementary Fig. 8 shows a graphical representation). Using biotinylated primers during PCR, DNA files can be labelled with biotin, which can be stably localized inside proteinosomes via the biotin–Tamavidin 2-HOT interaction. Different DNA-encoded files can be localized inside other proteinosomes to easily create multiple distinct localized files. **b**, After localization, files do not exchange between proteinosomes; therefore, multiple files can be pooled into a single library. This library can be amplified using multiplex PCR without molecular crosstalk and recovered using magnetic separation after PCR. Additionally, fluorescently barcoded libraries can be sorted into separate populations using fluorescence-assisted sorting.

Here we introduce a methodology, termed ‘thermoconfined PCR’, which employs microreactors with temperature-dependent membrane permeabilities to augment the fidelity of PCR amplification. Using this strategy, we implement multiplex, repeated PCR-based access of multiple DNA files from a complex DNA pool. Our method is based on stably encapsulating biotinylated DNA files in individual populations of thermoresponsive, semipermeable microcapsules (Fig. 1a) followed by the pooling of these populations. The membrane’s unique thermoresponsive permeability greatly reduces molecular crosstalk and the accompanying artifact formation at PCR temperatures, thereby allowing multiple data-encoded DNA files to be faithfully amplified, comparable with emulsion PCR. However, in contrast to emulsion PCR, non-biotinylated amplicons can be retrieved and separated from the original data-encoded DNA files at room temperature without destroying the microcompartment because membrane permeability is restored at room temperature (Fig. 1b).

Thermoconfined PCR uses proteinosomes, semipermeable microcompartments based on protein–polymer conjugates^36,37^. Biotinylated DNA files can be stably localized inside the proteinosome lumen using the encapsulated biotin-binding protein Tamavidin 2-HOT (Fig. 1a). We first demonstrate the heat-stable retention of biotin-labelled DNA templates inside Tamavidin 2-HOT proteinosomes. Next, we analyse the temperature-dependent permeability of proteinosomes for single-stranded DNA (ssDNA) and double-stranded DNA (dsDNA) using confocal fluorescence microscopy and find that membrane permeability significantly decreases at higher temperatures. Drawing on this observation, we determined that isolating individual reactions inside proteinosomes markedly reduces molecular crosstalk during multiplex PCR amplification of the two templates. These results were then scaled to the simultaneous amplification of 25 DNA files, totalling over 1.5 million unique sequences. We show that compared with bulk amplification, the multiplex amplification of this complex DNA pool leads to a more even sequence representation when reactions are localized inside proteinosomes. Additionally, we establish that proteinosomes can enable multiple repetitive read operations by co-encapsulating magnetic beads that allow the efficient recovery of the original encapsulated DNA files after PCR-based random access. As a final step, we confirm that our platform is compatible with fluorescent metadata tagging using short DNA strands and membrane labels. Further, we show that combined with fluorescence sorting, DNA files can be selectively retrieved and amplified based on metadata, an approach that has recently been used for similarity^38^ and Boolean file searches^39^.

## Results

### Heat-stable and temperature-responsive DNA localization

We have previously^37,40,41^ shown that streptavidin-containing proteinosomes can be prepared by covalently crosslinking bovine serum albumin (BSA)^42,43^/poly(*N*-isopropylacrylamide) (PNIPAm) conjugates at the interface of water-in-oil emulsion droplets. The crosslinked proteinosomes were phase transferred into water, and the addition of biotin-functionalized or dsDNA strands led to DNA template accumulation and stable retention up to 200 base pairs (bp) (ref. ^37^). In previous work^37^, we have characterized the proteinosome membrane and lumen in detail and observed the long-term and homogeneous co-localization of streptavidin and internalized biotinylated dsDNA.

**Fig. 2:**
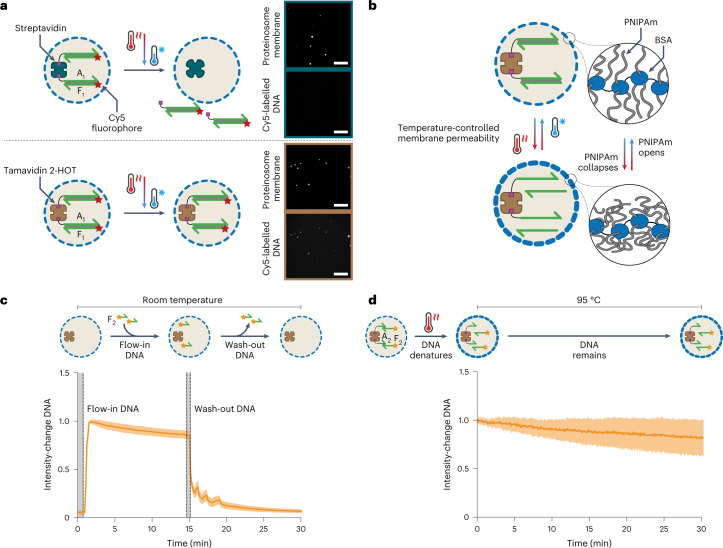
Temperature-dependent DNA localization. **a**, Confocal micrographs of DNA-containing proteinosomes after heating to 95 °C and cooling. Here dsDNA (188 bp; **A**_**1**_**F**_**1**_) with Cy5 and biotin labels was localized in DyLight-405-labelled proteinosomes containing either 4 µM streptavidin (top two panels) or Tamavidin 2-HOT (bottom two panels), heated to 95 °C for 5 min, and then cooled to room temperature before imaging. Micrographs of the proteinosome membrane and Cy5-labelled DNA are shown at the top and bottom of each panel, respectively. Only the proteinosomes containing Tamavidin 2-HOT retained dsDNA. Scale bars, 250 µm. Larger versions of these micrographs are shown in Supplementary Fig. 9. The sequences for strands **A**_**1**_ and **F**_**1**_ are provided in Supplementary Table 1. **b**, Graphic showing temperature-dependent, reversible proteinosome membrane collapse and opening due to PNIPAm chain collapse and swelling, respectively, when conjugated to crosslinked BSA (blue). High (*T* < LCST) and low (*T* > LCST) membrane permeabilities are shown as the thin or thick blue dashed circles, respectively. **c**, Relative fluorescence intensity of fluorophore-labelled DNA inside proteinosomes at room temperature as a function of time. Proteinosomes (*n* = 21) were confined in a microfluidic trapping array^37^ to enable simultaneous imaging and reagent addition or removal. A 50-nt-long ssDNA (**F**_**2**_) labelled with Alexa 546 fluorophore was added to the trapping array and diffusion across the membrane was measured using confocal microscopy. After the fluorescent signal stabilized, a buffer was added to remove DNA from the trapping chamber. The solid line indicates the mean signal; the shaded area indicates the standard deviation. The sequence of strand **F**_**2**_ is provided in Supplementary Table 1. **d**, Relative fluorescence intensity of fluorophore-labelled DNA inside proteinosomes at 95 °C as a function of time. The proteinosomes (*n* = 15) contained a dsDNA complex (*T*_m_ = 65 °C) consisting of 21 nt biotin-labelled DNA strand **A**_**2**_ and 50 nt Alexa-546-labelled strand **F**_**2**_. Proteinosomes were heated to 95 °C and the fluorescence was measured by confocal microscopy. The solid line indicates the mean signal; the shaded area indicates the standard deviation. The sequences for strands **A**_**2**_ and **F**_**2**_ are provided in Supplementary Table 1. Source data

To understand how their thermal properties influence the PCR of encapsulated DNA files, we investigated proteinosomes’ temperature-dependent stability and permeability. Since streptavidin^37,40,41^ is only partially resistant to the high temperatures used during PCR, we used the heat-stable streptavidin analogue Tamavidin 2-HOT^44^, which binds biotin with affinity similar to streptavidin but can tolerate the higher temperatures during PCR. We prepared proteinosomes containing either 4 µM streptavidin or 4 µM Tamavidin 2-HOT to verify both proteins’ heat stability therein (Methods). A homogeneous distribution of Tamavidin 2-HOT inside the lumen was verified (Supplementary Fig. 1) and the size of Tamavidin 2-HOT-containing proteinosomes was determined to be 57 ± 17 µm (mean ± standard deviation; Supplementary Fig. 2a). The 188-nucleotide (nt)-long, biotinylated, Cy5-labelled dsDNA **A**_**1**_**F**_**1**_ was then localized inside the prepared proteinosomes by incubating them with dsDNA (Methods and Supplementary Fig. 3). Confocal micrographs of proteinosomes revealed that after being heated to 95 °C, only proteinosomes prepared with Tamavidin 2-HOT retain the internalized dsDNA (Fig. 2a). To simplify the downstream retrieval of proteinosomes, we also incorporated superparamagnetic particles inside the proteinosomes (Methods). Together, these two changes generate proteinosomes containing heat-stable localized DNA coupled with magnetic retrieval.

As PNIPAm becomes partially immiscible above its lower critical solution temperature (LCST; ~32 °C), we reasoned that lowering the membrane permeability above the LCST could retain DNA molecules produced during the PCR-based processing of the captured DNA files (Fig. 2b). Previous work^36^ has shown that heating proteinosomes above the LCST reduces membrane permeability for hydrophobic molecules, possibly by increasing the membrane hydrophobicity and decreasing the pore size. To investigate temperature-dependent permeability for ssDNA, we first verified that proteinosomes containing encapsulated Tamavidin 2-HOT are permeable to ssDNA at room temperature. Using a previously^37^ developed microfluidic trapping array (Supplementary Fig. 4), we captured Tamavidin 2-HOT-containing proteinosomes and added a 50 nt Alexa-546-labelled ssDNA (**F**_2_) to the trapping chamber. As expected, ssDNA readily diffused across the membrane. Because ssDNA **F**_2_ is not biotinylated, washing the trapped proteinosomes results in a rapid loss of fluorescence, indicating that the proteinosome membrane is highly permeable to ssDNA at room temperature (Fig. 2c). Next, we prepared Tamavidin 2-HOT proteinosomes containing a biotinylated 21 nt strand **A**_**2**_ base-paired to a 10 nt section of Alexa-546-labelled 50 nt ssDNA **F**_**2**_ (predicted melting temperature, *T*_m_ = 65 °C) and heated the proteinosomes to 95 °C to ensure that the DNA duplex melted completely. Confocal imaging experiments at this temperature (Methods) revealed that localized fluorescence slowly decreased over time (Fig. 2d). This result, which could be attributed to either a small amount of Alexa-546-labelled DNA diffusing across the membrane or imaging artifacts (Supplementary Fig. 5), indicates that membrane permeability to relatively long ssDNA is much lower at this temperature. In a similar experiment at 95 °C using a shorter 31 nt Alexa-546-labelled ssDNA (similar in length to most primers), DNA rapidly diffused across the membrane (Supplementary Fig. 6). Once cooled to temperatures below the LCST, the membrane was again highly permeable to both long and short ssDNA (Supplementary Fig. 7), suggesting that the temperature-induced change in membrane permeability is reversible. Together, our results reveal that Tamavidin 2-HOT proteinosomes stably localize the biotinylated dsDNA at higher temperatures even after the DNA duplex melts and that the membrane’s high permeability is restored on cooling to temperatures below the LCST.

### Enzymatic amplification of proteinosome-localized DNA

**Fig. 3:**
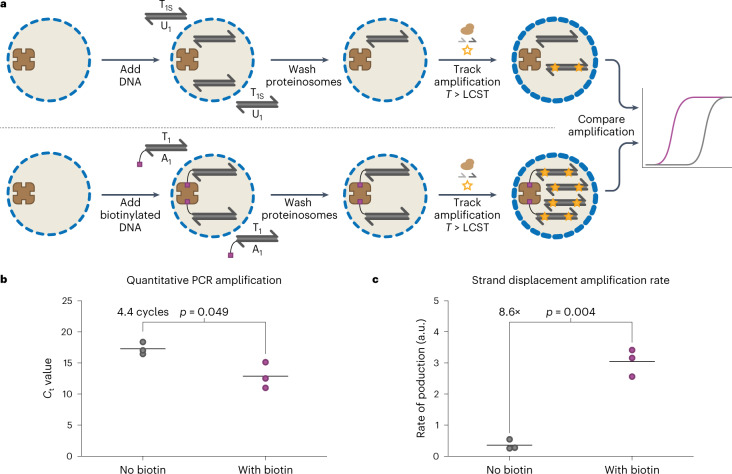
Enzymatic amplification of DNA localized inside proteinosomes. **a**, Experimental design to measure the enzymatic amplification of localized DNA templates inside proteinosomes containing 4 µM Tamavidin 2-HOT. DNA strands with (178 bp; **A**_**1**_**T**_**1**_) or without (168 bp; **U**_**1**_**T**_**1S**_) a biotin end modification were added to the proteinosomes and removed with five washing steps (Methods). An amplification reaction mixture containing enzymes, primers, dNTPs and dsDNA-specific EvaGreen dye was then added and the amplification of DNA localized in the proteinosomes was measured using real-time fluorescence monitoring. States of high (*T* < LCST) and low (*T* > LCST) membrane permeability are shown as thin or thick blue dashed circles, respectively. **b**, qPCR results of amplified DNA from proteinosomes incubated with either biotin-labelled DNA or non-labelled DNA (Methods). The horizontal lines indicate the mean threshold cycle (*C*_t_) for three experiments; the points represent individual experiments. A statistically significant difference of 4.4 cycles between the two conditions is observed using a two-sided Welch’s *t*-test (*p* = 0.049). Individual amplification traces are shown in Supplementary Fig. 11. The sequences used are listed in Supplementary Table 2. **c**, SDA results of isothermally amplifying DNA from proteinosomes incubated with biotin-labelled DNA or non-labelled DNA (Methods). The horizontal lines denote the mean production rate for three experiments; the points represent individual experiments. A statistically significant 8.6-fold difference in rates is observed between the two conditions using a two-sided Welch’s *t*-test (*p* = 0.004). Individual amplification traces are shown in Supplementary Fig. 12. The sequences used are listed in Supplementary Table 2. Source data

Having established that (1) biotinylated DNA remains localized inside Tamavidin 2-HOT proteinosomes on heating to PCR temperatures, (2) both strands of a long duplex with a single biotin modification remain localized inside the proteinosomes even when the duplex melts and (3) membrane permeability can be controlled by temperature, we proceeded to enzymatically amplify the localized DNA templates. Previously^8,45,46^, both PCR and strand displacement amplification (SDA) have been used to amplify data-encoding DNA molecules (Supplementary Fig. 10). To demonstrate the general applicability of proteinosomes containing internalized Tamavidin 2-HOT for DNA data retrieval, we next show that localized biotinylated DNA with a length typically used for data storage can be amplified using either PCR or SDA (Fig. 3a).

To measure localized dsDNA amplification by PCR in situ, we used quantitative PCR (qPCR; Methods). Proteinosomes containing 4 µM Tamavidin 2-HOT were incubated with 300 nM of either biotinylated 178 nt template dsDNA **T**_**1**_**A**_**1**_ or non-biotinylated 168 nt control dsDNA **T**_**1S**_**U**_**1**_. After washing to remove the free template, we added a PCR reaction mixture consisting of polymerase, deoxynucleoside triphosphates (dNTPs), primers and the dsDNA-sensitive fluorescent dye EvaGreen. For each reaction, the threshold cycle was determined (Methods) to assess the amount of DNA accessible for amplification. On average, 4.4 fewer cycles were needed to reach the fluorescent threshold when biotinylated dsDNA was used instead of non-biotinylated dsDNA (Fig. 3b). Assuming perfect amplification during each PCR cycle, this indicates that around 21 times more dsDNA is available for amplification when dsDNA is biotinylated compared with the background levels, demonstrating that proteinosome-localized dsDNA is accessible for PCR amplification.

Next, we used SDA to isothermally amplify localized dsDNA. As shown above, we incubated proteinosomes containing 4 µM Tamavidin 2-HOT with 300 nM of either biotinylated template dsDNA **T**_**1**_**A**_**1**_ or unlabelled **T**_**1S**_**U**_**1**_ before washing and then adding an amplification mixture consisting of polymerase, ssDNA-binding protein, nickase, dNTPs, primers and EvaGreen. We employed dsDNA-sensitive EvaGreen to track DNA production, but since SDA is a linear process, we extracted the production rates from the fluorescence data (Methods). The SDA reactions of proteinosomes containing **T**_**1**_**A**_**1**_ showed, on average, an 8.6 times higher amplification rate than proteinosomes incubated with non-biotinylated **T**_**1S**_**U**_**1**_ (Fig. 3c). Together, the results obtained with PCR and SDA indicate that biotinylated dsDNA localized by Tamavidin 2-HOT and with lengths typically used for DNA data storage can be amplified from within proteinosomes.

### Thermoconfined multiplex PCR ameliorates molecular crosstalk

**Fig. 4:**
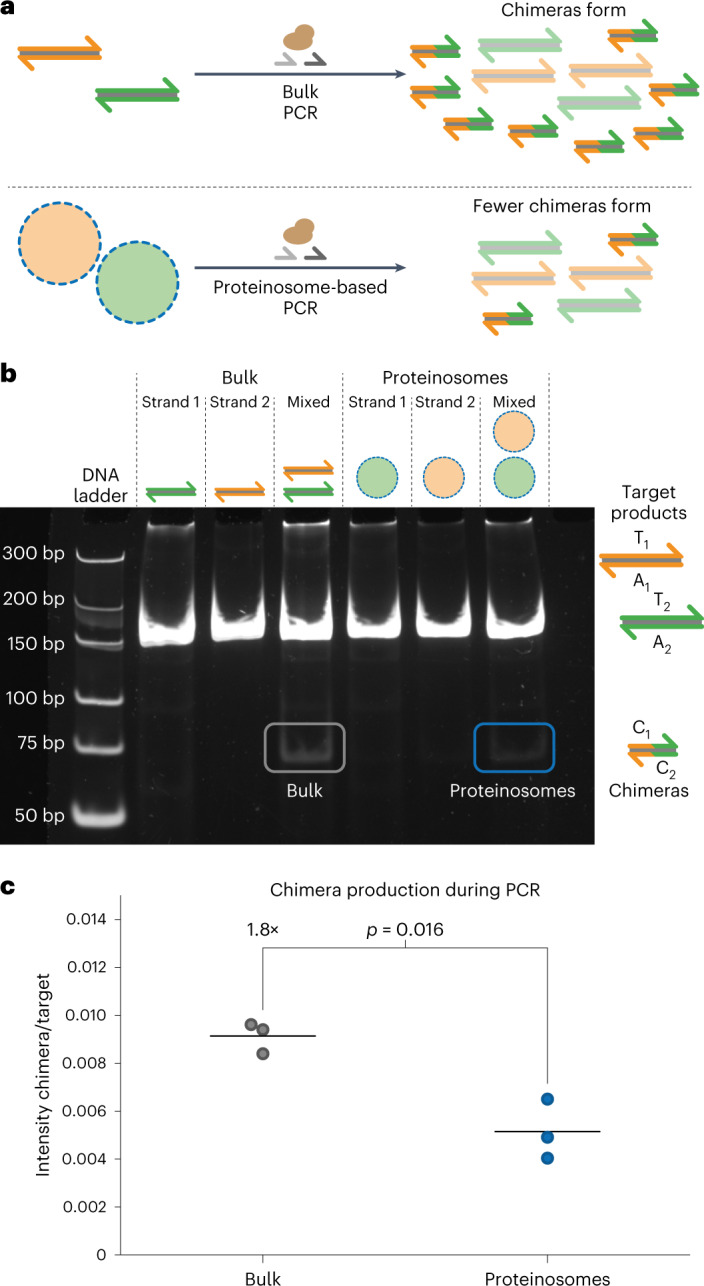
Localized amplification reduces molecular crosstalk during PCR. **a**, Thermoconfined PCR within proteinosomes reduces chimera formation during multiplex PCR by limiting molecular crosstalk. **b**, Representative native PAGE gel of the reaction mixtures. Unpurified mixtures of simplex and duplex PCR in bulk or within proteinosomes were loaded on a native PAGE gel and stained using SYBR Gold before visualization. Target strands are 178 bp and designed such that chimeras of 71 bp are expected to form. Full-length 178 bp product formation occurs in all the samples, whereas chimera formation is mostly observed in duplex PCR samples. The sequences used are listed in Supplementary Table 3. **c**, Quantitative analysis of PAGE results. Duplex PCR reactions were independently repeated three times and analysed using native PAGE. The intensities of the target and chimera bands were determined for each reaction (Methods); the target-to-chimera ratios for different amplification methods are shown. The horizontal lines indicate the mean ratio between the chimera and target intensities; the data points indicate three individual experiments. A statistically significant 1.8-fold difference is observed using a two-sided Welch’s *t*-test (*p* = 0.016). Individual gel analyses are shown in Supplementary Fig 13. The sequences used are listed in Supplementary Table 3. Source data

PCR-based random access can retrieve encoded data from complex DNA pools^8^. However, sequences that contain highly similar short regions are susceptible to recombination during PCR^30^. The formation of such chimeric amplicons corrupts DNA files because data are inserted at incorrect positions. Additionally, chimera formation leads to PCR bias in complex pools of DNA^30^, which has previously been inhibited by employing emulsion PCR^35,47,48^. We reasoned that proteinsomes’ temperature-controlled membrane permeability should reduce chimera formation during the multiplex PCR of complex DNA pools since the templates are effectively segregated at typical PCR temperatures (Fig. 4a). To investigate how thermoconfined, multiplex PCR from proteinosome-localized templates influences chimera formation, we designed two sets of biotinylated 178-nt-long dsDNA templates (**T**_**1**_**A**_**1**_ and **T**_**2**_**A**_**3**_) that share a 31 nt complementary region such that chimera **C**_**1**_**C**_**2**_ formed during multiplex PCR is 71 bp long (Fig. 4b). To localize the two templates inside separate proteinosomes containing 4 µM Tamavidin 2-HOT, we added 300 nM dsDNA and then washed the microcapsules to remove the excess DNA. Proteinosome populations were mixed and amplified using multiplex PCR (Methods) and amplicons were recovered at room temperature. As a control experiment, templates were also amplified in a bulk reaction. Native polyacrylamide gel electrophoresis (PAGE) analysis (Fig. 4b and Methods) revealed the formation of a chimera product, namely, **C**_**1**_**C**_**2**_, with a length of 71 bp, in the bulk reaction. Thermoconfined multiplex PCR also showed a faint band at 71 bp, but this band has a considerably lower intensity compared with the bulk PCR. Both reactions produced approximately the same amount of target DNA, as judged by the intensity of the bands at 178 bp. To quantify the effect of thermoconfinement during multiplex PCR, we utilized Fiji^49^ to measure the intensities of the target and chimera bands (Methods and Supplementary Fig. 13). We used the target band intensity as an internal control to account for gel-to-gel variability and calculated the chimera-to-product ratio for each reaction (Fig. 4c). Bulk reactions yielded higher chimera-to-target ratios, indicating that significantly more chimera is formed under bulk conditions compared with proteinosome-localized amplification. Some formation of chimeric DNA was observed during multiplexed PCR using proteinosomes, which can be attributed to the incomplete removal of non-localized DNA or limited release of amplicons from the proteinosomes. Even though complete chimera suppression as expected for emulsion PCR was not observed^35^, this result demonstrated that thermoconfined multiplex PCR in proteinosomes significantly reduces chimera formation by localizing DNA amplification to individual compartments.

### Multiplex and repeated PCR of localized DNA files

**Fig. 5:**
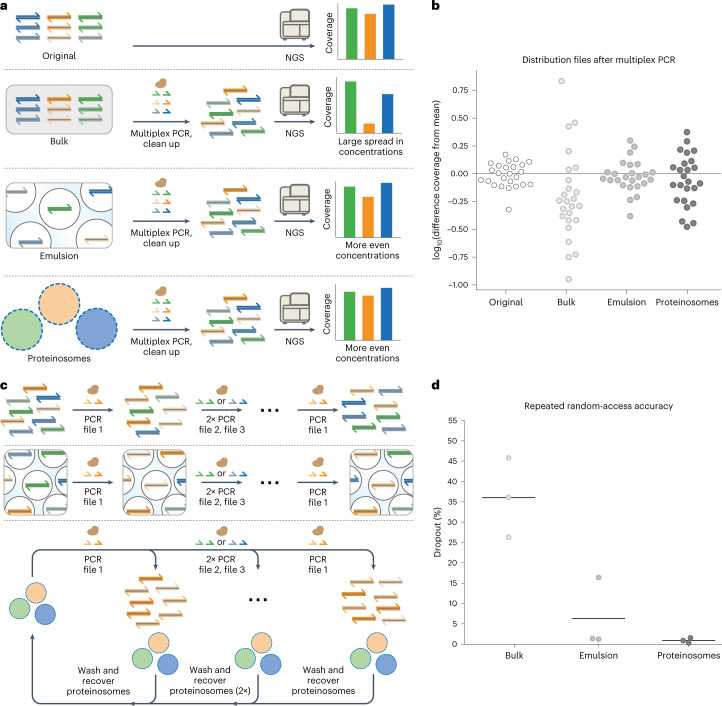
Multiplex PCR and repeated access of DNA-encoded files within proteinosomes. **a**, Schematic of the experiments used to determine the effect of localization on DNA file concentration distributions after PCR amplification in bulk or within either water-in-oil emulsion droplets or proteinosomes in water. Multiplex PCR was used to amplify twenty-five 1 MB DNA-encoded files, totalling 25 MB. Purified reaction mixtures were subsequently sequenced using Illumina sequencing and aligned to reference sequences to determine per-file coverage (Methods). NGS, next-generation sequencing. **b**, Scatter plot showing the log_10_ change from mean coverage per amplification method. Each point shows the log_10_ deviation from the mean coverage for an individual file. We observed 3-fold, 60-fold, 5-fold and 7-fold changes between the most and least sequenced files for reference, bulk, emulsion and thermoconfined amplification, respectively. The primer sequences used are listed in Supplementary Table 4. **c**, Schematic of the repeated random DNA access experiment. In three consecutive PCR reactions, the files were amplified using part of the previous reaction. After three files were randomly accessed, the first amplified file was accessed again using PCR and the reaction mixtures were purified. Using Illumina sequencing, we determined the dropout per file after the final PCR (Methods). **d**, Sequence dropout in the final file amplified after repeated-access PCR, as determined using Illumina sequencing (Methods) and aligned to the reference sequences (Methods), randomly sampled to ×30 coverage for direct comparison. The horizontal lines indicate the mean dropout of the final file accessed after four rounds of PCR for three different repeated random-access orders, and the points indicate individual data points. The histograms showing individual coverage distributions of the sequencing reads are shown in Supplementary Fig. 15. The sequences used are listed in Supplementary Table 4. Source data

Having shown that spatially segregating PCR reactions reduces chimera formation, we reasoned that localizing data-encoded files inside Tamavidin 2-HOT proteinosomes should better maintain file representation since chimera formation negatively influences PCR bias^30^. To demonstrate this, twenty-five 1 MB files, altogether totalling 25 MB, are stored in DNA and encoded into 110-base DNA sequences using a previously reported method^8^. Each file, consisting of approximately 66,000 unique sequences, was localized inside individual Tamavidin 2-HOT proteinosome populations. We then pooled 25 populations together to create a proteinosome-based library. Next, the files were amplified using multiplex PCR in the bulk solution or compartmentalized media comprising either water-in-oil emulsion droplets or proteinosome populations in water (Methods). The relative file concentrations after multiplex PCR were quantified using qPCR (Methods) to determine the fraction of each file (Supplementary Fig. 14). The qPCR data revealed that thermoconfined and emulsion PCR preserved the file distribution more effectively than bulk amplification. We then performed Illumina sequencing (Fig. 5a) to determine the average coverage per file (Methods and Supplementary Table 5) to test if improved proportionality translated into more homogeneous and even coverage per file. The average coverage per file normalized to the mean was plotted on a logarithmic scale to show the coverage deviation in orders of magnitude (Fig. 5b and Methods). As expected, we observed a large 60-fold difference in coverage between the most and least represented files amplified under bulk conditions. In contrast, the files amplified using localized reactions showed much smaller spreads of seven- and fivefold differences for proteinosome- and emulsion-based PCR, respectively. The initial spread present in the library before amplification was threefold. Additionally, we determined the coefficients of variation (CVs) in sequencing coverage for all the conditions as a measure of distribution evenness. The CVs of the original pool, bulk-amplified DNA, emulsion droplets and proteinosomes were 24%, 139%, 35% and 52%, respectively. These results show that thermoconfined PCR is a viable alternative to oil-based emulsions for the proportional multiplex amplification of DNA-encoded data.

Since biotinylated DNA remains localized inside Tamavidin 2-HOT proteinosomes, we anticipate that recovering the proteinosomes, as well as their encapsulated DNA-encoded files, using magnetic separation after PCR enables reliable repeated access to DNA-encoded data. To test this, three files were localized inside proteinosomes containing 4 µM Tamavidin 2-HOT. The proteinosomes were washed before pooling to create a proteinosome-based library. Additionally, the files were also mixed in equimolar concentrations in the bulk to generate a non-localized library. From the resulting libraries, the files were amplified in three successive rounds of bulk, emulsion or proteinosome PCR, each round retrieving a different file. After three rounds, we again accessed the initial file (Methods). Importantly, between the PCR reactions, we recovered the proteinosome-localized library using magnetic separation (Methods) and reused the library for amplification of the next file. The library amplified using bulk PCR was purified by inactivating dNTPs and primers before being used for the amplification of the next file (Methods). Finally, the library amplified using emulsion PCR was purified by breaking the emulsion followed by spin column purification (Methods). Using Illumina sequencing, we determined the dropout within a file, meaning the fraction of sequences expected to be part of the file but no longer observed (Fig. 5c and Methods). We observed that repeatedly accessing the proteinosome-based library PCR yielded the lowest loss of sequences, followed by emulsion PCR and bulk PCR (Fig. 5d). Since sequence dropout can alternatively be addressed using error correction codes, we performed a quantitative analysis to show how the reduced dropout affects the coding rate (Supplementary Note 1). Our analysis shows that when the dropout rate changes from 36.10% to 0.91%, as observed from bulk to proteinosome PCR, respectively, the density increases by a factor of 1.56 times. Comparing the CVs of amplified DNA revealed that the spread in sequence coverages followed a similar trend as the dropout (mean bulk CV, 219%; mean emulsion CV, 96%; mean proteinosome CV, 69%). Together, these results demonstrate that the proteinosome-based encapsulation of DNA files and subsequent pooling of large DNA libraries simplify reliable repeated multiplex PCR operations.

### Fluorescence-based proteinosome-localized DNA file retrieval

**Fig. 6:**
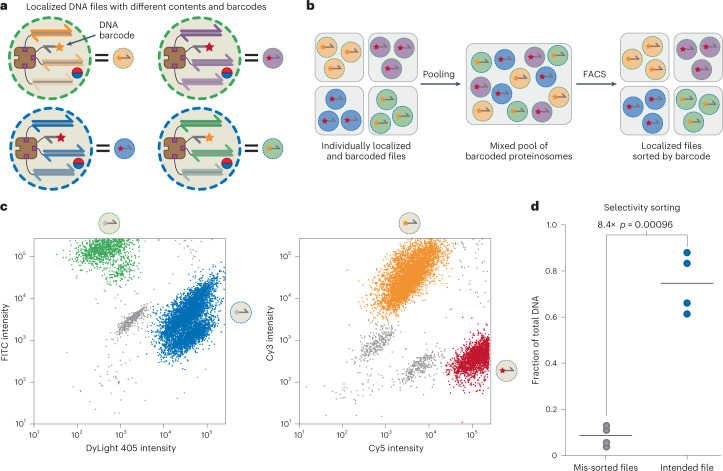
Fluorescence-assisted sorting of proteinosomes for selective file retrieval. **a**, Schematic of membrane- and localized DNA-based barcoding of proteinosomes. The proteinosome membranes are labelled with either DyLight 405 (blue) or FITC (green); additionally, fluorescent biotinylated ssDNA labelled with Cy3 (orange; **F**_**4**_) or Cy5 (red; **F**_**5**_) can be localized inside the proteinosomes alongside the DNA files. **b**, Individual, barcoded populations of localized files can be pooled together in a single searchable library. Proteinosomes are sorted via FACS based on the four barcodes (Methods). Effective sorting was verified using qPCR. **c**, FACS dot plots of FITC versus DyLight 405 (left) and Cy3 versus Cy5 (right) labels. Both plots contain two distinct clusters that were used to sort the pooled proteinosomes into four populations. Supplementary Fig. 16 shows the full gating strategy and histograms of individual fluorescent channels. Sequences for **F**_**4**_ and **F**_**5**_ are listed in Supplementary Table 5. **d**, Sorting selectivity as determined by qPCR. The horizontal lines indicate the mean file fractions; the circles indicate three individual data points. Unintentionally mis-sorted files account for 8% of the total DNA concentration, whereas the intended file sorting accounts for 75% of the total. There is a statistically significant difference of 8.4-fold change in the relative fractions observed using a two-sided Welch’s *t*-test (*p* = 0.00096). Sequences for **F**_**4**_, **F**_**5**_ and the primers used are listed in Supplementary Table 6. Source data

The PCR-based retrieval of DNA files is very specific; however, the need for sufficiently orthogonal primers limits how many files can be stored in a single pool^8^. This limitation has led to the development of supplementary strategies for randomly accessing DNA files, such as physical separation^50^, hybridization-based retrieval^38^, alternative amplification schemes^33,51^ and fluorescence-assisted sorting^39,52^. In addition to demonstrating repeated multiplex PCR, we sought to devise methods for selectively retrieving files from DNA-encoded proteinosome libraries. To achieve this goal, we generated a strategy for fluorescently barcoding proteinosome populations based on (1) labelling the microcapsule membrane with either fluorescein isothiocyanate (FITC) and DyLight 405 and (2) adding short biotinylated, Cy3 (**F**_**4**_)-labelled or Cy5 (**F**_**5**_)-labelled ssDNA after the initial data-encoded DNA files have been localized inside the proteinosomes (Fig. 6a and Methods). This approach allows us to differentiate between four proteinosome populations, although, in theory, it is possible to generate up to 2^*N*^ unique barcode combinations, where *N* is the number of fluorophores used for barcoding. After encapsulating the data-encoding files in barcoded proteinosomes, we pooled the individual populations and used fluorescence-activated cell sorting (FACS) to select specific files from the pool (Fig. 6b) via a three-step selection procedure. First, proteinosomes were selected against unincorporated magnetic particles and background using gating on FSC-A and FSC-H channels (Supplementary Figs. 15 and 16). Second, the FITC and DyLight 405 membrane labels were used as fluorescence gates, as the fluorophores’ presence or absence produced distinct bimodal distributions in both channels (Fig. 5c (left) and Supplementary Fig. 16). Third, Cy3 and Cy5 fluorescence gates were established to distinguish between the localized **F**_**4**_ and **F**_**5**_ sub-populations (Fig. 6c (right) and Supplementary Fig. 16). Consequently, the pooled proteinosomes were sorted into four different populations based on their distinct fluorescence characteristics: (1) high DyLight 405/high Cy5; (2) high DyLight 405/high Cy3; (3) high FITC/high Cy3; and (4) high FITC/high Cy5. Following sorting, these four populations were evaluated using flow cytometry to determine the sorting accuracy (Supplementary Fig. 17). Only unimodal distributions were observed in the fluorescence data, thus indicating homogeneous populations. Having sorted the pool into individual populations, we used qPCR to determine the fractions of each file in the separated populations to confirm that the correct DNA-encoded data were retrieved (Fig. 6d). We found that the intended files account for, on average, 75.0% of all the DNA in the sorted samples (non-target files were, on average, 8.4% each), thereby demonstrating enrichment against other files in each sample. These results show that not only are proteinosomes compatible with PCR-based random access but the fluorescence-based metadata retrieval of proteinosomes is also possible.

### Lyophilization of DNA-containing proteinosomes

For archival DNA-based data storage, the long-term stability of DNA is critical. Previous work has shown that the lyophilization of DNA increases the hydrolytic stability of DNA by approximately one order of magnitude^53^. We performed the lyophilization of proteinosomes containing internalized DNA in the presence of trehalose, a lyoprotectant^54,55^ known to further enhance DNA stability^16,56^. Microscopy and qPCR experiments revealed that the rehydration of the solid powder did not affect DNA integrity and co-localization inside the proteinosomes (Supplementary Fig. 19). Importantly, we did not observe the coalescence of proteinosomes after rehydration.

## Conclusions

Advances in the synthesis and sequencing of DNA have enabled the long-term storage of digital data on DNA. However, repeated, and parallel, PCR-based random access has, thus far, been challenging. This study developed a methodology based on thermoresponsive, semipermeable microcompartments that enables multiplexed PCR-based random access with performances comparable with emulsion PCR. However, unlike in emulsion PCR, encapsulated DNA-encoded files remain localized inside the microcompartments after amplification and data retrieval, thereby enabling the repeated copying of the original file-encoding molecules. Additionally, we incorporated fluorescence-based barcodes and used these for proteinosome-based file sorting, providing an additional layer of data organization. At the current DNA synthesis prices, the use of proteinosomes for DNA file localization would not lead to a substantial increase in price per MB of stored data (Supplementary Note 2), whereas the physical density of proteinosome-localized data is comparable with other DNA encapsulation methods (Supplementary Note 3). Compared with emulsion PCR, the initial localization of DNA-encoded data files based on the proteinosome platform is relatively time-consuming; however, once localized, the encapsulated files are fully accessible for repeated, PCR-based amplification without considerable processing steps, simplifying data retrieval (Supplementary Note 4). Additionally, we successfully demonstrated the lyophilization and subsequent rehydration of proteinosome-localized DNA files. Future studies will be aimed at investigating the long-term stability of encapsulated DNA in the dried state using accelerated ageing assays^56^.

In this study, data retrieval from proteinosome-localized files was realized by amplifying DNA from many similar file-containing proteinosomes, which negatively affects the data density, as a single proteinosome, in principle, contains sufficient DNA copies for reliable file decoding^12^. Another limitation of the current study is the use of FACS for the fluorescence-based retrieval of proteinosome-localized DNA files. Although FACS has the advantage of fast and selective file retrieval, it results in a considerable loss of encapsulated files and does not allow for the retrieval of a single compartment. As a solution, we envision the magnetic retrieval of single encapsulated files by integrating magnetic, fluorescently labelled proteinosomes with electromagnetic microneedle technology^57^ and automated fluorescence microscopy. Similarly, a reduction in the polydispersity of current proteinosome microcapsules by employing a microfluidic^58^ production platform would also benefit the retrieval of proteinosome-localized DNA files. These improvements provide a path towards the repeated, parallel random access of extremely dense proteinosome-based DNA file libraries, which are compatible with a broad range of data-encoding and DNA amplification methods.

## Methods

### Materials

2-Ethyl-1-hexanol (98%, Sigma); 1-(3-dimethylaminopropyl)-3-ethylcarbodiimide HCl (Carbosynth); 1,6-diaminohexane (98%, Sigma); PEG-bis-(*N*-succinimidyl succinate) (*M*_w_ = 2,000, Sigma); DyLight 405 NHS ester (ThermoFisher); FITC NHS ester (ThermoFisher); BSA (heat-shock fraction, pH 7.0, ≥98%; Sigma); streptavidin from *Streptomyces avidinii* (Sigma); Tamavidin 2-HOT, recombinant (Wako Chemicals); Dynabeads M-270 amine (Invitrogen); Dynabeads MyOne carboxylic acid (Invitrogen); 1 M MgCl_2_ (Invitrogen); 1 M Tris pH 8.0 RNase free (Invitrogen); 5 M NaCl (Invitrogen); EvaGreen (Biotium); KAPA HiFi HotStart PCR kit (Roche); Micellula DNA emulsion and purification kit (EURx); ibidi anti-evaporation oil (ibidi); 30% (19:1 monomer:bis) acrylamide solution (Bio-Rad); and SYBR Gold (ThermoFisher) were used as received. All the other chemicals used were purchased from Sigma. The enzymes were purchased from New England Biolabs, unless noted otherwise.

### Synthesizing BSA-NH_2_–PNIPAm nanoconjugates

Cationized BSA (BSA-NH_2_) was synthesized according to a previously reported method^36^. Typically, a solution of diaminohexane (1.5 g, 12.9 mmol in 10 ml Milli-Q water) was adjusted to pH 6.5 using 5 M HCl and added dropwise to a stirred solution of BSA (200 mg, 3 μmol in 10 ml Milli-Q water). The coupling reaction was initiated by adding 100 mg of 1-(3-dimethylaminopropyl)-3-ethylcarbodiimide HCl immediately and then another 50 mg after 5 h. If needed, the pH value was readjusted to 6.5 and the solution was stirred for another 6 h and then centrifuged to remove any precipitate. The supernatant was dialysed (Medicell dialysis tubing; molecular weight cutoff (MWCO) of 12−14 kDa) overnight against Milli-Q water and freeze dried.

End-capped mercaptothiazoline-activated PNIPAm (*M*_n_ = 13,284 g mol^−1^, 4 mg in 5 ml Milli-Q water) was synthesized according to the previously reported method^1^ and added to a stirred solution of BSA-NH_2_ (10 mg in 5 ml PBS buffer at pH 8.0). The molecular weight and polydispersity of activated PNIPAm were determined using gel permeation chromatography (Supplementary Fig. 20). The solution was stirred for 10 h and then purified using a centrifugal filter (Millipore, Amicon Ultra; MWCO, 50 kDA) and freeze dried. After freeze drying, the obtained BSA-NH_2_–PNIPAm conjugate was characterized by matrix-assisted laser desorption/ionization–mass spectrometry and zeta potentiometry (Supplementary Fig. 20).

FITC- and DyLight-405-labelled BSA-NH_2_–PNIPAm conjugates were prepared using the same method, except that labelled BSA was used as the starting material.

### Labelling BSA with fluorescent dyes

BSA was labelled with FITC as follows: 200 mg of BSA was dissolved in 10 ml of 50 mM sodium carbonate buffer (pH 9). Then, 2.36 mg FITC was dissolved in 590 μl DMSO and added to the stirred BSA solution. The resulting solution was stirred for 5 h, purified by dialysing (Medicell dialysis tubing; MWCO, 12−14 kDa) overnight against Milli-Q water and freeze dried. BSA was labelled with DyLight 405 as follows: 30 mg of BSA was dissolved in 6 ml of 50 mM sodium carbonate buffer (pH 9). Then, 1 mg of DyLight 405 NHS ester was dissolved in 100 μl DMF and added to the stirred BSA solution. The solution was stirred for 2 h, purified by dialysing (Medicell dialysis tubing; MWCO, 12−14 kDa) overnight against Milli-Q water and freeze dried. Using ultraviolet–visible spectrophotometry, we determined the average number of dyes per protein. For DyLight 405 labelling, we measured, on average, 1.4 dyes per BSA molecule. For FITC labelling, we measured 1.3 dyes per BSA molecule.

### Preparing Tamavidin 2-HOT-containing proteinosomes

Proteinosomes containing Tamavidin 2-HOT and magnetic particles (Dynabeads M-270 amine) were prepared similar to previous^37^ descriptions for streptavidin-containing proteinosomes. Proteinosomes used for the experiments (Fig. 1c–e) did not contain magnetic particles. BSA–PNIPAm nanoconjugates (6 mg ml^–1^ total, 1 mg ml^–1^ of which was fluorescently labelled) were mixed with 4 µM Tamavidin 2-HOT and 4 mg ml^–1^ Dynabeads in 7.5 µl aqueous phase; 0.6 mg of PEG-bis(*N*-succinimidyl succinate) (*M*_w_ = 2,000) was dissolved in 7.5 µl of 50 mM sodium carbonate buffer (pH 9) and added to the mix, which was then briefly vortexed. Next, 300 µl of 2-ethyl-1-hexanol was added, followed by vortexing to yield the Pickering emulsion. The resulting mixture was left at room temperature for 2 h to crosslink the nanoconjugates. The oil phase was removed by pipetting away the upper oil layer and 300 µl of 70% ethanol was added to resuspend the sediment. The dispersion was then sequentially dialysed (Medicell dialysis tubing; MWCO, 12−14 kDa) against 70% and 50% ethanol for 2 h each and finally overnight against Milli-Q water to yield proteinosomes in water. Proteinosomes were then stored at 4 °C for later use.

### DNA oligonucleotide synthesis

Except DNA encoding files from Twist Bioscience, all the DNA oligonucleotides were purchased from Integrated DNA Technologies. Modified oligonucleotides that were purified with high-performance liquid chromatography were purchased, and desalted non-modified oligonucleotides were purchased. Stock solutions (100 and 10 μM) were made using nuclease-free TE buffer (10 mM Tris, 0.1 mM EDTA, pH 7.5; Integrated DNA Technologies) and stored at −30 °C. DNA encoding for the files was ordered from Twist Bioscience. These files were individually PCR amplified in a 20 µl reaction containing 1 ng DNA pool, 0.5 µM forward and reverse primers, and KAPA HiFi HotStart polymerase. The amplification protocol was as follows: denaturing at 95 °C for 3 min, denaturing at 98 °C for 20 s, annealing at 65 °C for 15 s and extending at 72 °C for 15 s. The second denaturing, annealing and extending steps were repeated 8–10 times, followed by a final extension at 72 °C for 30 s before cooling down to 4 °C. The resulting amplicons were then purified using the Qiagen PCR extraction kit following the manufacturer’s instructions. The files that were obtained this way were then mixed in equal ratios in 10 ng and shipped at room temperature from Seattle to Eindhoven. These templates were then used similar to ssDNA oligonucleotides ordered from Integrated DNA Technologies.

### Preparing dsDNA with biotin of fluorophores

Double-stranded complexes consisting of strands shorter than 100 nt were formed by thermal annealing. Biotinylated strands were mixed with non-biotinylated strands at 12 and 10 µM and heated to 95 °C in a thermocycler for 3 min. The samples were subsequently cooled to room temperature at a rate of −0.5 °C min^–1^.

Here dsDNA strands longer than 100 bp, with fluorescent and/or biotin modifications, were prepared from either single-stranded ultramer templates or dsDNA files and modified primers using PCR since these constructs could not be directly ordered. Typically, reactions were performed at the 100 µl scale using 5 µl (1 nM) diluted template, primers (0.5 µM each) and KAPA HiFi HotStart polymerase. The amplification protocol was as follows: denaturing at 95 °C for 3 min, denaturing at 98 °C for 20 s, annealing at 65 °C for 15 s and extending at 72 °C for 15 s. The second denaturing, annealing and extending steps were repeated 16 times, followed by a final extension at 72 °C for 30 s before cooling down to 4 °C. The resulting amplicons were then purified using the Qiagen PCR extraction kit following the manufacturer’s instructions.

### Localizing DNA in proteinosomes

Biotin-labelled DNA was initially localized in 10.0 mM Tris (pH 8.0) with 11.5 mM MgCl_2_ and 0.1% vol/vol Tween 20. Typically, 10 µl of proteinosome-containing solution was added to 5 µl of 4× buffer solution and 5 µl of DNA to be localized. The mixture was kept at 4 °C overnight. The following day, 500 µl wash buffer consisting of 10.0 mM Tris (pH 8.0), 1 M NaCl, 11.5 mM MgCl_2_ and 0.1% vol/vol Tween 20 was added, left at 4 °C overnight and removed the following day. Secondary washing steps were performed similar to the first steps, except that no overnight step was used. Instead, proteinosomes were separated from the solution by placing the mixture in a magnetic separation rack (DynaMag, Invitrogen) for 3 min, after which the supernatant was removed by a pipette.

### Initial localization stability testing

Proteinosomes containing localized DNA were heated to 95 °C in a 10 µl solution using a MiniPCR thermocycler for at least 5 min. After cooling to room temperature, a 2 µl drop was placed on a glass microscopy coverslip and confocal micrographs were taken.

### Temperature-dependent fluorescence microscopy

Fluorescence data were acquired using a confocal laser scanning microscope (Leica SP8) equipped with solid-state lasers (405 nm for DyLight 405, 552 nm for Alexa 546 and 638 nm for Cy5) and a hybrid detector in the photon-counting mode. The time-lapse measurements were performed with a ×10/0.40 numerical aperture (field of view, 1.55 × 1.55 mm^2^; slice thickness, 7 μm) at a resolution of 512 × 512 pixels. High-temperature confocal laser scanning microscopy data were obtained using a VAHEAT micro-heating system (Interherence) with SmS-r substrates. A 50 µl solution of DNA-loaded proteinosomes was pipetted onto the sample cell and covered with 150 µl ibidi anti-evaporation oil to prevent evaporation. Room-temperature diffusion experiments were conducted in our previously^37^ described microfluidic trapping array. Data processing was done using a custom Python code similar to what we have previously described^37^.

### Statistics

All the results reporting statistical values were obtained from independent triplicates. The analysis was performed using Python’s SciPy (Python 3.6.5, SciPy version 1.1.0) library. Welch’s two-sided *t*-test was used to compare two populations. Statistical significance between more than two samples was determined using one-way analysis of variance, followed by a post-hoc analysis using Tukey’s multiple comparison testing. Only values of *p* < 0.05 were considered to be statistically significant.

### SDA

The SDA of DNA localized in proteinosomes was performed using a protocol adapted from another work^46^. The reaction mixture consisted of 1× NEB buffer 2, 0.5 µM primer, 250.0 µM dNTP each, 0.125 U µl^–1^ Klenow Fragment (Exo-), 0.250 U µl^–1^ Nt.BspQI, 0.2 mg ml^–1^ BSA, 4 µM T4 gene 32 protein and 1× EvaGreen. The reaction volume was 25 µl, 2 µl of which consisted of proteinosomes in a buffer (10.0 mM Tris with 11.5 mM MgCl_2_ and 0.1% vol/vol Tween 20). The reaction was kept at 37 °C and recorded using a CFX96 Touch real-time PCR detection system (Bio-Rad). To prevent evaporation, the plate was sealed with a transparent sticker. The production rate was determined using Python by fitting a linear function to the fluorescence intensity and cycle number.

### qPCR

qPCR was performed using the CFX96 Touch real-time PCR detection system (Bio-Rad). The total reaction volume was 25 µl and consisted of KAPA HiFi HotStart, 0.5 µM primers, 1× EvaGreen and 2 µl template solution (DNA or proteinosomes). The initial denaturation was set to 3 min at 95 °C, and then 40 denaturation cycles at 98 °C for 20 s, annealing at 65 °C for 15 s and extension at 72 °C for 15 s were performed, followed by a final extension at 72 °C for 30 s before cooling down to 4 °C. Fluorescence was measured during each annealing step. To prevent evaporation, the plate was sealed with a transparent plate sealer. CFX Maestro software version 3.1.1517.0823 (Bio-Rad) was used to perform baseline correction and calculate the threshold cycles (*C*_t_).

### Chimera formation determination using PAGE gel analysis

The total reaction volume was 25 µl and consisted of KAPA HiFi HotStart, 0.5 µM primers and 2.5 µl proteinosome solution. Thermocycling was performed in a T1000 Touch thermocycler (Bio-Rad). Following initial denaturation for 3 min at 95 °C, 20 cycles of denaturation at 98 °C for 20 s, annealing at 65 °C for 15 s and extension at 72 °C for 15 s were performed, followed by a final extension at 72 °C for 30 s before cooling down to 4 °C. To the PCR mixtures, 5 µl of 6× loading dye (ThermoFisher) was added before loading 12 µl on 10% TB-Mg PAGE gels. The gels were cast using 30% (19:1 monomer:bis) acrylamide solution. Running and gel buffers were 44.5 mM Tris, 44.5 mM boric acid and 11.5 mM MgCl_2_. The gels were run for 1 h 15 min at 150 V in a Criterion vertical cell electrophoresis device (Bio-Rad) and stained for 10–15 min using SYBR Gold (ThermoFisher). The images were taken using an ImageQuant 400 Digital Imager (GE Healthcare). Bands-of-interest image intensities were determined using ImageJ’s gel analysis plug-in. In some gels, a signal is observed at the top of the gel, which we attribute to larger complexes (such as polymerase–DNA complexes) present in unpurified reaction mixtures. These signals were not considered in the analysis. Statistical analysis was performed using Python.

### Emulsion PCR

Emulsion PCR of the library was performed using a Micellula DNA emulsion and purification kit. A 50 µl reaction mixture containing 25 µl KAPA HiFi HotStart 2×, 50 ng template DNA, 4 µM primers (0.16 µM per pair) and 1.25 mg ml^–1^ BSA was used. The emulsion was formed by adding 300 µl premixed inorganic phase and vortexing at the maximum speed in a fridge at 4 °C for 5 min, per the manufacturer’s instructions. The resultant emulsion was split into four tubes and thermocycled as follows: initial denaturation for 3 min at 95 °C, 18 cycles of denaturation at 95 °C for 20 s, annealing at 65 °C for 15 s and extension at 72 °C for 15 s were performed, followed by a final extension at 72 °C for 30 s before cooling down to 4 °C. The reactions were pooled, the emulsion was broken and DNA was purified according to the manufacturer’s instructions.

The relative concentrations of individual files in the purified DNA were then quantified using qPCR.

### Quantifying multiplex PCR concentration

DNA localized inside a mixed pool of proteinosomes or in bulk was amplified in 25 µl reactions consisting of 2 µl template DNA and primers (0.4 µM of each forward and reverse pair; Supplementary Table 4 lists the primer sequences) using KAPA HiFi HotStart polymerase. Thermocycling was performed in a C1000 Touch thermocycler (Bio-Rad). Following initial denaturation for 3 min at 95 °C, 18 cycles of denaturation at 98 °C for 20 s, annealing at 65 °C for 15 s and extension at 72 °C for 15 s were performed, followed by a final extension at 72 °C for 30 s before cooling down to 4 °C. The reaction mixtures were purified using the Qiagen PCR extraction kit following the manufacturer’s instructions.

The relative concentrations of individual files in the purified DNA were then quantified using qPCR.

### Library preparation and sequencing

Multiplex PCR reactions were performed at the Eindhoven University of Technology and shipped at room temperature to the University of Washington. On receipt, the samples were validated using an Implen nanophotometer. Subsequently, the samples were prepared for sequencing following the Illumina TruSeq Nano DNA Library Prep protocol. The ends were blunted with the End Repair buffer (ERP2) and then purified with Beckman Coulter AMPure XP beads; an ‘A’ nucleotide was annealed to the 3’ end with an A-tailing ligase. Ligation was performed using Illumina sequencing adapters from Illumina’s TruSeq DNA CD Indexes kit, with each sample ligated to a unique Illumina index. Finally, the samples were cleaned using Illumina sample purification beads and enriched using a 12 cycle PCR. The final product length and purity were qualified using a QIAxcel Bioanalyser. Then, the samples were individually quantified using qPCR and mixed to create an equal-mass library.

A final library was prepared for sequencing by following the Illumina NextSeq Denature and Dilute Libraries Guide. The sequencing libraries were loaded in the Illumina NextSeq at 1.3 pM with a 20% control spike-in of the ligated PhiX genome.

### Analysis of Illumina sequencing data

Basecalling and demultiplexing of the sequenced samples were performed using bcl2fastq. The generated FASTQ files were then aligned against the reference sequences using Burrows–Wheeler Aligner^59^. Next, the coverage for each sequence was determined using SAMtools^60^.

### Repeated-access PCR in bulk

Three files were mixed in equal molar ratios to a final concentration of 0.5 nM. Three aliquots were amplified in 100 µl reactions consisting of 5 µl template mix and primers (0.5 µM) using KAPA HiFi HotStart polymerase. The PCR cycling protocol comprised initial denaturation for 3 min at 95 °C, 10 cycles of denaturation at 98 °C for 20 s, annealing at 65 °C for 15 s and extension at 72 °C for 15 s, followed by a final extension at 72 °C for 30 s before cooling down to 4 °C. A 5 µl aliquot was taken in which primers and dNTPs were then inactivated using Exo-CIP Rapid PCR Cleanup Kit (NEB). From the resulting mixture, 5 µl was used in the next PCR as a template. The remaining 95 µl of the PCR reaction was stored at −30 °C.

### Repeated access using emulsion PCR

Three files were mixed in equal molar ratios and emulsion PCR of the library was performed using a Micellula DNA emulsion and purification kit. A 50 µl reaction mixture containing 25 µl KAPA HiFi HotStart 2×, 50 ng template DNA, 0.5 µM primers and 1.25 mg ml^–1^ BSA. The emulsion was formed by adding 300 µl premixed inorganic phase and vortexing at the maximum speed in a fridge at 4 °C for 5 min, per the manufacturer’s instructions. The resultant emulsion was split into four tubes and thermocycled as follows: initial denaturation for 3 min at 95 °C, 10 cycles of denaturation at 95 °C for 20 s, annealing at 65 °C for 15 s and extension at 72 °C for 15 s were performed, followed by a final extension at 72 °C for 30 s, before cooling down to 4 °C. The reactions were pooled, the emulsion was broken and DNA was purified according to the manufacturer’s instructions. From this purified reaction, 1 µl was used in the next reaction.

After four rounds of repeated access, the final purified mixture was amplified using bulk PCR to generate enough DNA for sequencing experiments. The protocol for this amplification used a total reaction volume of 25 µl and consisted of KAPA HiFi HotStart, 0.5 µM primers and 2 µl purified reaction mixture. Thermocycling was performed in a T1000 Touch thermocycler (Bio-Rad). Following initial denaturation for 3 min at 95 °C, 20 cycles of denaturation at 98 °C for 20 s, annealing at 65 °C for 15 s and extension at 72 °C for 15 s were performed, followed by a final extension at 72 °C for 30 s before cooling down to 4 °C. The reaction mixture was then purified using the Qiagen PCR extraction kit following the manufacturer’s instructions.

### Repeated-access PCR in proteinosomes

Three files were localized in individual proteinosome populations, washed five times and mixed to create the final pool. Three aliquots were amplified in 100 µl reactions consisting of 5 µl of the proteinosome library and primers (0.5 µM), using KAPA HiFi HotStart polymerase. The PCR cycling protocol comprised initial denaturation for 3 min at 95 °C, 10 cycles of denaturation at 98 °C for 20 s, annealing at 65 °C for 15 s and extension at 72 °C for 15 s, followed by a final extension at 72 °C for 30 s before cooling down to 4 °C. The reaction mixtures were then placed on a magnetic separation rack (DynaMag, Invitrogen) for 3 min to recover the proteinosomes. Next, 95 µl of the reaction mixture was pipetted off and stored at −30 °C. The remaining 5 µl was washed three times using 100 µl wash buffer (10.0 mM Tris (pH 8.0), 1 M NaCl, 11.5 mM MgCl_2_ and 0.1% vol/vol Tween 20). After the magnetic recovery of proteinosomes, a new reaction mix was added to the washed 5 µl solution of proteinosomes to make a final volume of 100 µl.

### Preparing Tamavidin 2-HOT-containing proteinosomes for sorting

Due to the smaller sizes required by the nozzle used and interference from the magnetic particles normally employed, proteinosomes utilized in the sorting experiment were prepared according to a slightly modified protocol. Proteinosomes containing Tamavidin 2-HOT and magnetic particles (Dynabeads MyOne carboxylic acid) were prepared using methods similar to those described above. BSA–PNIPAm nanoconjugates (6.00 mg ml^–1^ total, 1.00 mg ml^–1^ of which were fluorescently labelled) were mixed with 4 µM Tamavidin 2-HOT and 0.75 mg ml^–1^ Dynabeads in 20 µl total aqueous phase. Next, 0.6 mg of PEG-bis(*N*-succinimidyl succinate) (*M*_w_ = 2,000) was dissolved in 40 µl of 50 mM sodium carbonate buffer (pH 9), added to the mix and briefly vortexed. Then, 1 ml 2-ethyl-1-hexanol was added and the mixture was subsequently vortexed for 30 min to yield the Pickering emulsion. The resulting mixture was left at room temperature for 1.5 h to crosslink the nanoconjugates. The oil phase was removed by pipetting away the upper oil layer and 500 µl of 70% ethanol was added to resuspend the sediment. The dispersion was then sequentially dialysed (Medicell dialysis tubing; MWCO, 12−14 kDa) against 70% and 50% ethanol for 2 h each and finally overnight against Milli-Q water to yield proteinosomes in water, before filtering using a 30 µm CellTrics cell strainer (Sysmex). We verified these proteinosomes’ reduced size using confocal microscopy, the results of which are shown in Supplementary Fig. 2. Proteinosomes were then stored at 4 °C for later use.

### Fluorescence-assisted sorting

A FACS Aria III flow cytometer (BD Biosciences), operating at low–middle pressure, was used to interrogate a mix of proteinosomes through a 100 μm nozzle. DyLight-405- and FITC-labelled proteinosomes were prepared for FACS, and the files encoded in DNA were localized overnight in the proteinosomes. These proteinosomes were subsequently washed with 500 µl wash buffer (10.0 mM Tris (pH 8.0), 1 M NaCl, 11.5 mM MgCl_2_ and 0.1% vol/vol Tween 20) and stored overnight at 4 °C. The next day, 500 µl supernatant was removed, and fluorescently barcoded DNA (labelled with biotin and either Cy5 or Cy3) was added to the proteinosomes and allowed to localize for 15 min at room temperature. After barcode localization, the proteinosomes were washed four more times by the addition of 500 µl wash buffer, followed by magnetic separation and supernatant removal. Proteinosome populations were mixed just before sorting. A total of 1,000–2,000 events were recorded, from which two-dimensional plots of the forward-scattered light height (FSC-H) versus forward-scattered light area (FSC-A) were obtained. FITC fluorescence was interrogated using a 488 nm laser and a 530/30 nm detector; Dylight 405 was excited at 405 nm and detected at 460/55 nm. Cy5 (ex, 633; em, 660/20). Cy3 (ex, 561; em, 582/15). The gating was performed with BD FACSDiva software (BD Biosciences) and consisted of initial gating in the FSC-H versus FSC-A plot to select the proteinosomes against background and unincorporated magnetic beads. From this initial gate, we defined a high FITC and high DyLight 405 gate, each of which were subsequently split into high Cy3 and high Cy5 gates. These were the final gates used to sort the proteinosomes. Supplementary Fig. 16 shows the detailed gating strategy. Sorting was performed in BSA-coated tubes and the sorted populations were reanalysed using BD Aria III flow cytometer, without sorting. The final graphs were plotted using flowCore and flowViz packages in R.

## Online content

Any methods, additional references, Nature Portfolio reporting summaries, source data, extended data, supplementary information, acknowledgements, peer review information; details of author contributions and competing interests; and statements of data and code availability are available at 10.1038/s41565-023-01377-4.

## Supplementary information

Supplementary Information Supplementary Figs. 1–21, Notes 1–4, Tables 1–6, Unprocessed gels 1–4 and methods.

Supplementary Data DNA sequences of oligonucleotides used throughout the study; each tab contains sequences belonging to a specific figure.

## Data Availability

The following data are available via Zenodo at 10.5281/zenodo.7553674: AutoCAD drawing of the microfluidic trapping device, sequences of the DNA used to encode the 25 files used in the current study and FASTQ files of the sequencing experiments (Fig. 5b,d). Any other data are available from the corresponding authors upon reasonable request. Source data are provided with this paper.

## Source data

Source Data Fig. 2 Raw data points of all the graphs presented in Fig. 2. Data are ordered with a separate tab for the figure and correspondingly labelled.
Source Data Fig. 3 Raw data points of all the graphs presented in Fig. 3. Data are ordered with a separate tab for the figure and correspondingly labelled.
Source Data Fig. 4 Raw data points of all the graphs presented in Fig. 4. Data are ordered with a separate tab for the figure and correspondingly labelled as well as for the unprocessed PAGE gel of the chimeric PCR experiment. The processed version is shown in Fig. 4b.
Source Data Fig. 5 Raw data points of all the graphs presented in Fig. 5. Data are ordered with a separate tab for the figure and correspondingly labelled.
Source Data Fig. 6 Raw data points of all the graphs presented in Fig. 6. Data are ordered with a separate tab for the figure and correspondingly labelled.

## Code availability

Python scripts that allow for the reproduction of our sequencing data analysis are available via Zenodo at 10.5281/zenodo.7553674.
