# Supplementary material for: DNA storage in thermoresponsive microcapsules for repeated random multiplexed data access
Source: Nat Nanotechnol. 2023 May 4;18(8):912–21. doi: 10.1038/s41565-023-01377-4 (PMC10427423; doi:10.1038/s41565-023-01377-4)

nature nanotechnology

Article

https://doi.org/10.1038/s41565-023-01377-4

DNA storage in thermoresponsive microcapsules for repeated random multiplexed data access

In the format provided by the authors and unedited

Contents

Supplementary Methods and Materials 2

Preparation of fluorescent Tamavidin 2-HOT 2

SDS-PAGE 2

Confocal microscopy 2

Brightfield and epifluorescence microscopy 2

Determining Tamavidin 2-HOT loading capacity 2

Determining DNA loading capacity Tamavidin 2-HOT proteinosomes 3

Gel Permeation Chromatography 3

MALDI-MS 3

Zeta potentiometry 3

Characterisation size of Tamavidin 2-HOT containing proteinosomes and emulsion droplets 3

Lyophilization of proteinosomes 4

Supplementary Figures 5

Supplementary Figure 1 Measuring Tamavidin 2-HOT loading. 5

Supplementary Figure 2 Sizes of compartments used for localized PCR. 6

Supplementary Figure 3 Determination dsDNA loading capacity. 7

Supplementary Figure 4 Microfluidic trapping array design. 8

Supplementary Figure 5 Detailed images belonging to figure 2d. 9

Supplementary Figure 6 Diffusion of 31mer ssDNA over proteinosome membranes is not affected by temperature 9

Supplementary Figure 7 Reversibility of membrane collapse. 10

Supplementary Figure 8 Graphical representation of a general DNA file 10

Supplementary Figure 9 Larger versions of micrographs in figure 2a 11

Supplementary Figure 10 Cartoons of DNA amplification methods used. 12

Supplementary Figure 11 Individual traces qPCR experiment summarized in Fig. 3b. 12

Supplementary Figure 12 Individual traces of strand displacement amplification experiment summarized in Fig. 3c. 13

Supplementary Figure 13 Data analysis individual gels Fig. 4d. 13

Supplementary Figure 14 PCR inside physically separated compartments maintain the original spread of concentrations in DNA library 14

Supplementary Figure 15 Coverage distributions after repeated PCR. 15

Supplementary Figure 16 Flow cytometry analysis comparing beads and proteinosomes 15

Supplementary Figure 17 Gating strategy used to sort four proteinosome populations. 16

Supplementary Figure 18 Results post-sort flow cytometry. 17

Supplementary Figure 19 Assessing proteinosome stability during lyophilization. 18

Supplementary Figure 20 Characterization proteinosome components. 18

Supplementary Figure 21 Size distribution proteinosomes used for FACS after filtering. 19

Supplementary Notes 20

Supplementary Note 1 Effect dropout on coding density 20

Supplementary Note 2 Estimation of price per MB 20

Supplementary Note 3 Estimation of storage density and latency 20

Supplementary Note 4 Complexity comparison DNA-file compartmentalization methods 21

Supplementary Tables 23

Supplementary Table 1 – DNA sequences used in Figure 2 23

Supplementary Table 2 – DNA sequences used in Figure 3 23

Supplementary Table 3 – DNA sequences used in Figure 4 24

Supplementary Table 4 – DNA sequences used in Figure 5 25

Supplementary Table 5 – Coverage per file for each condition of plot in figure 5b 27

Supplementary Table 6 – Sequences used in figure 6 27

Unprocessed gels 30

Unprocessed gel relating to Supplementary Figure 1 30

Unprocessed gel relating to Supplementary Figure 13 – 1 31

Unprocessed gel relating to Supplementary Figure 13 – 2 32

Unprocessed gel relating to Supplementary Figure 13 - 3 33

Supplementary Methods and Materials

Preparation of fluorescent Tamavidin 2-HOT

Fluorescent Tamavidin 2-HOT was prepared by coupling of Alexa647-NHS (Thermofisher) to Tamavidin 2-HOT. In a 20 µL reaction, 50 µM Tamavidin 2-HOT in PBS was reacted with 1 mM Alexa647-NHS dissolved in DMSO, the final DMSO concentration was 12.5 v/v%. The mixture was allowed to react for 2 hours in the dark and subsequently, excess dye was removed using Zeba Micro Spin Desalting Columns, 7K MWCO (Thermofisher). Conjugate concentration was determined using NanoDrop. Using SDS-PAGE (Supplementary Fig. 1) it was determined that after purification there was still 69% unreacted dye present.

SDS-PAGE

For SDS-PAGE analysis 4-20% SDS-PAGE Mini-PROTEAN® TGX Precast gels (Bio-rad) were used. The running buffer consists of 25 mM Tris, 192 mM glycine, 0.1% SDS, pH 8.3. Samples were heated at 95 °C for 5 min in 1x SDS Sample Buffer (62.5 mM Tris, 10% Glycerol, 2.5% SDS (w/v), 0.01% Bromophenol blue, pH 6.8) before loading. The gels were run at 150 V for 40 minutes. Photographs of gels were taken using an ImageQuant 400 Digital Imager (GE Healthcare). Fluorescence intensities of bands of interest were determined using ImageJ's gel analysis plug-in.

Confocal microscopy

Quantitative fluorescence data were acquired using a confocal laser scanning microscope (CLSM, Leica SP8) equipped with solid-state lasers (405 nm for DyLight405, 488 nm for FITC, 552 nm for Alexa546, 638 nm for Cy5) and a hybrid detector (DyLight405 emission was measured ata 415nm - 448nm, FITC at 498nm - 528nm, Alexa546 at 562nm - 602nm and Cy5 at 656nm - 696nm). The time-lapse measurements were performed with a ×10/0.40 numerical aperture (NA) (1.55 × 1.55 mm2 field of view, 7 µm slice thickness) at a resolution of 512 × 512 pixels. The photon-counting mode of the hybrid detector was used. High-temperature CLSM data was obtained using VAHEAT-Micro heating system (Interherence GmbH) with SmS-r substrates. A 50 µL sample solution was pipetted onto the sample cell and covered with 150 µL Ibibidi anti-evaporation oil to prevent evaporation. Time course experiments at room temperature were conducted in our previously described microfluidic trapping array[1], data processing was done using custom Python code, similarly to our previously described method[1]. Single confocal micrographs were made by placing a 1 µL droplet of sample on a glass cover slide.

Brightfield and epifluorescence microscopy

All brightfield and epifluorescence microscopy was performed at room temperature. Imaging was performed using a Leica SP8 microscope using a ×10/0.40 numerical aperture (NA). An external light source (Leica) was used for epifluorescence imaging. To visualize DyLight405 fluorescence the DAPI fluorcube was used, FITC fluorescence was detected using FITC fluorcube. Images were captured using the attached digital camera on a field of view 1.2421x0.9313 mm^2 and a resolution of 960x720 pixels.

Determining Tamavidin 2-HOT loading capacity

The encapsulation efficiency of Tamavidin 2-HOT inside proteinosomes was determined by preparing proteinosomes as normal with AF647-labelled Tamavidin 2-HOT. These proteinosomes were imaged using confocal microscopy and the internal concentration of AF647-labelled Tamavidin 2-HOT was determined by comparing fluorescence intensity against a calibration series. Any non-reacted dye was assumed to have been removed during the overnight dialysis of proteinosomes but was present in the calibration series, which we corrected for this using previously measured labelling efficiency. To prepare a calibration curve, 1 µL drops of known concentrations AF647-Tamavidin 2-HOT were placed on a glass slide and three confocal micrographs were taken at different Z-positions. Mean fluorescence intensities were determined using LASX software (Leica GmbH).

Determining DNA loading capacity Tamavidin 2-HOT proteinosomes

Loading capacity for DNA inside proteinosomes was determined by incubating proteinosomes containing 4µM Tamavidin 2-HOT with 300 nM biotin- and Cy5 labelled dsDNA in 10 mM Tris (pH 8.0) with 11.5 mM $MgCl_2$ and 0.1% vol/vol Tween-20. 10 µL of the proteinosome-containing solution was added to 5µL 4x buffer solution and 5 µL of DNA to be localized. The mixture was kept at 4°C overnight and the following day 500 µL wash buffer consisting of 10 mM Tris (pH 8.0) 1 M NaCl, 11.5 mM $MgCl_2$ and 0.1% vol/vol Tween-20 was added and left at 4°C overnight and removed the following day. Four secondary washing steps were performed similarly to the first except that no overnight step was used, instead proteinosomes were separated from the solution by placing the mixture in a magnetic separation rack (DynaMag, Invitrogen) for three minutes, after which the supernatant was removed by pipette. These proteinosomes were imaged using confocal microscopy and the internal concentration of Cy5-labelled DNA was determined by comparing fluorescence intensity against a calibration series. To prepare a calibration curve, 1 µL drops of biotin- and Cy5-labelled DNA of known concentrations were placed on a glass slide and three confocal micrographs were taken at different Z-positions. Mean fluorescence intensities were determined using LASX software (Leica GmbH).

Gel Permeation Chromatography

Polymer molecular weight distributions were measured using mixed-C and mixed-D columns (Polymer Labs, 300 x 70 mm) connected in series at 40°C using a Shimadzu Prominence-i system. The eluent was tetrahydrofuran (THF) and the concentration of polymer was 1.0 mg/mL. The data were processed using Shimadzu GPC software.

MALDI-MS

MALDI-TOF MS was performed on an Autoflex Speed instrument (Bruker, Bremen, Germany) using 2,5-dihydroxybenzoic acid as matrix substance. The sample aqueous solution concentration was 1.0 mg/mL.

Zeta potentiometry

Zeta potentiometry measurements were done using a Malvern ZetaSeizer Nano ZSP. Samples (0.1 mg/ml) were measured in PBS (pH 7.4 0.1 mM) buffer at room temperature. Data were analysed using Malvern ZetaSeizer software and plotted using Python.

Characterisation size of Tamavidin 2-HOT containing proteinosomes and emulsion droplets

The size distribution of proteinosomes and emulsion droplets was determined using fluorescence and brightfield microscopy. A 2 µL droplet containing proteinosomes or emulsion PCR droplets was placed on a glass coverslip and a micrograph was taken. The diameter per proteinosome was determined using FIJI/ImageJ, the resulting distributions are shown in Supplementary Fig. 2.

Lyophilization of proteinosomes

Proteinosomes were lyophilized in standard wash buffer (10 mM Tris, 11.5 mM $MgCl_2$, 1M NaCl) supplemented by 250 mM trehalose. The proteinosome-containing mixture was snap-frozen by submersion in liquid nitrogen and subsequently freeze-dried. Reconstitution of proteinosomes consisted of rehydrating the obtained dry matter in equal volume nuclease-free water.

Supplementary Figures

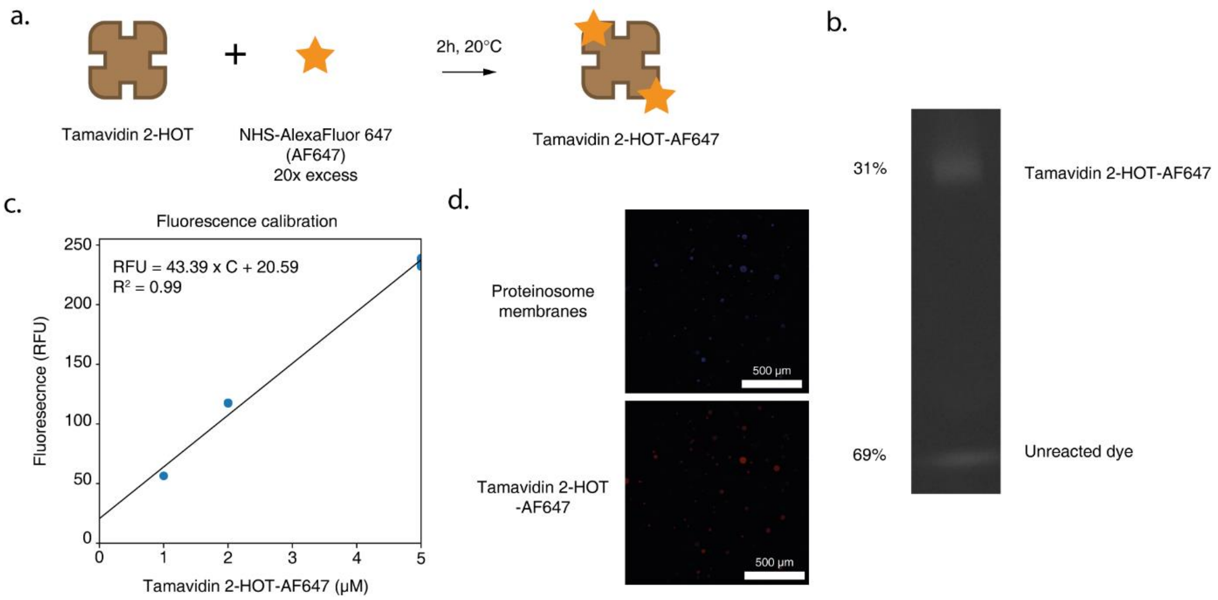

Supplementary Figure 1 Measuring Tamavidin 2-HOT loading. a. Fluorescent labelling strategy using NHS-AlexaFluor 647. **b.** Image of a non-reduced SDS-PAGE gel (4-20% gradient polyacrylamide, unstained) of the purified AF647-Tamavidin 2-HOT. Gel band intensity analysis was used to determine contamination of free AF647 (see Supplementary Methods). **c.** Calibration curve of fluorescence intensity vs concentration Tamavidin 2-HOT-AF647 determined at room temperature. **d.** Confocal micrographs of proteinosomes made using Tamavidin 2-HOT-AF647 at room temperature. Proteinosomes were prepared as normal, except fluorescently labelled Tamavidin 2-HOT was used. The final internal Tamavidin 2-HOT-AF657 concentration was determined using the calibration curve and accounted for the removal of unreacted dye by dividing by 0.31. The final concentration was measured to be 4.00±0.68 µM (*n=5*).

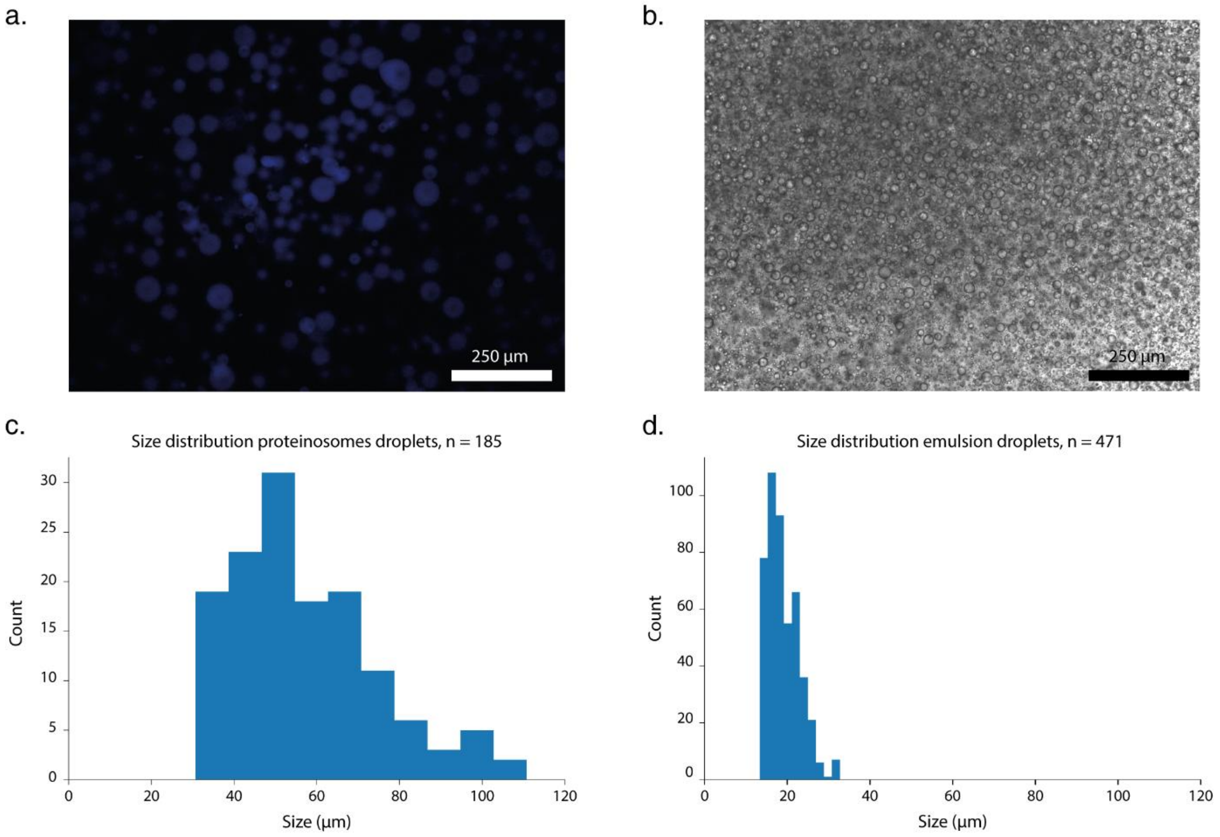

Supplementary Figure 2 Sizes of compartments used for localized PCR. a. Fluorescent micrograph of proteinosomes on a microscopy slide at room temperature. **b.** Brightfield micrograph of emulsion on a microscopy slide at room temperature. Emulsion PCR mixture was prepared as recommended by the manufacturer, except water was used instead of PCR reaction mixture and imaged using brightfield microscopy. **c.** Size distribution of proteinosomes. The micrograph shown in **a** was analysed using FIJI/ImageJ to determine the diameter of proteinosomes and plotted in a histogram. **d.** Size distribution of emulsion droplets. The micrograph shown in **b** was analysed using FIJI/ImageJ to determine the diameter of proteinosomes and plotted in a histogram.

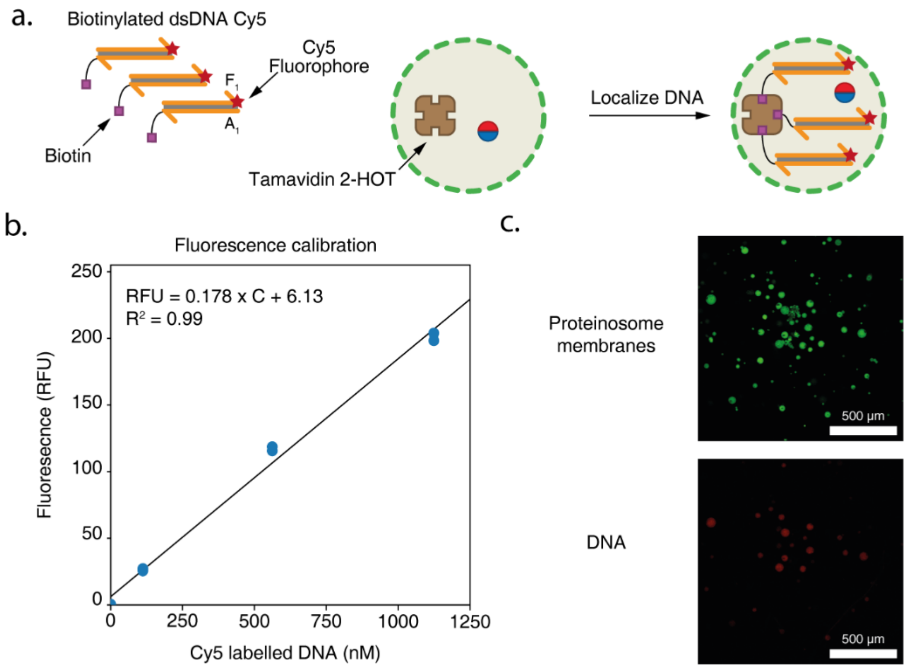

Supplementary Figure 3 Determination dsDNA loading capacity. a. Loading of fluorescent DNA inside Tamavidin 2-HOT proteinosomes. A 188 basepair (bp) dsDNA probe $\boldsymbol{A_1F_1}$ containing both a biotin and Cy5 label was prepared and localized inside FITC-labelled proteinosomes containing 4µM Tamavidin 2-HOT by incubating 300 nM DNA with proteinosomes overnight before washing and imaged using confocal microscopy at room temperature. **b.** Calibration curve of fluorescence intensity vs concentration Cy5- and biotin-labelled dsDNA determined at room temperature. **c.** Confocal micrographs of proteinosomes (green) made with 4µM Tamavidin 2-HOT containing localised Cy5- and biotin-labelled dsDNA (red). Fluorescence intensity of proteinosome subsections were determined and internalized dsDNA concentration was determined using a calibration curve. The final concentration of DNA was determined to be 93.8±12.7nM (*n=10*). Sequences for $\boldsymbol{A_1}$ and $\boldsymbol{F_1}$ are listed in Supplementary Table 1.

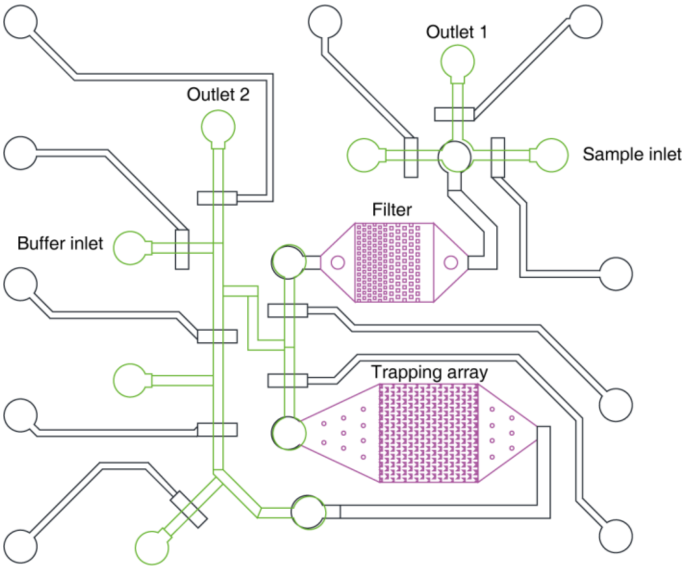

Supplementary Figure 4 Microfluidic trapping array design. CAD drawing of the microfluidic device, adapted from previously developed design[1,2]. Trapping array and filter are highlighted in purple. The channels of the bottom layer (bonded to the glass slide) are shown in black. The rounded top layer channels are shown in green. The flow channels crosses from the top layer to the bottom layer, as only the rounded channels of the top layer can be closed using pneumatic valves, while the trapping array requires the rectangular channels of the bottom layer. In a typical experiment proteinosomes or DNA are loaded via sample inlet port and flown through outlet 1 until no air bubbles were observed. Flow was then directed through the filter and trapping array to the outlet 2. If needed buffer could be added directly to the trapping array using the buffer inlet port.

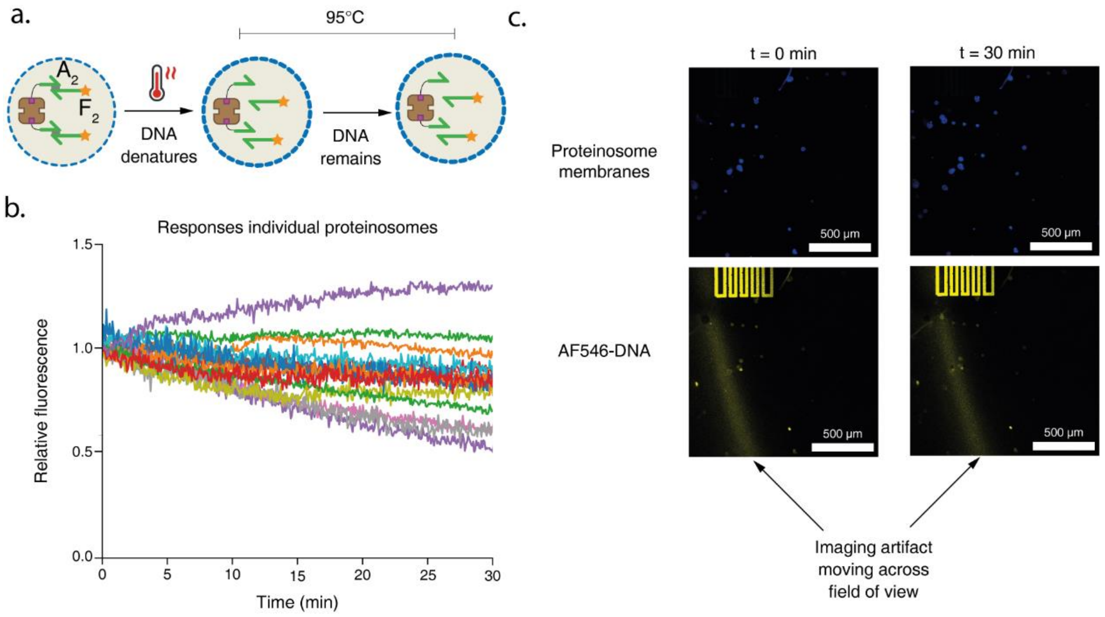

Supplementary Figure 5 Detailed images belonging to figure 2d. a. Cartoon depicting experimental setup, as described in main text Figure 2d. **b.** Relative fluorescence intensity of fluorophore-labelled DNA inside proteinosomes at 95˚C as a function of time. A double-stranded DNA complex (T_m=65°C) consisting of 21 nt biotin-labelled DNA strand (***A_2***) and 50 nt Alexa546 labelled strand (***F_2***) was localized inside proteinosomes (n=15). Proteinosomes are subsequently heated to 95°C while Alexa546 fluorescence was measured using confocal microscopy. Each trace is the mean fluorescence of an individual proteinosome relative to its fluorescence at t = 0 minutes. **c.** Confocal micrographs of proteinosomes containing AF546-labelled DNA in VAHEAT heating slides at times t=0 minutes and t=30 minutes. A reflection is observed to move across the imaging field of view in the AF546-DNA channel, complicating imaging. Sequences for ***A_2*** and ***F_2*** are listed in Supplementary Table 1.

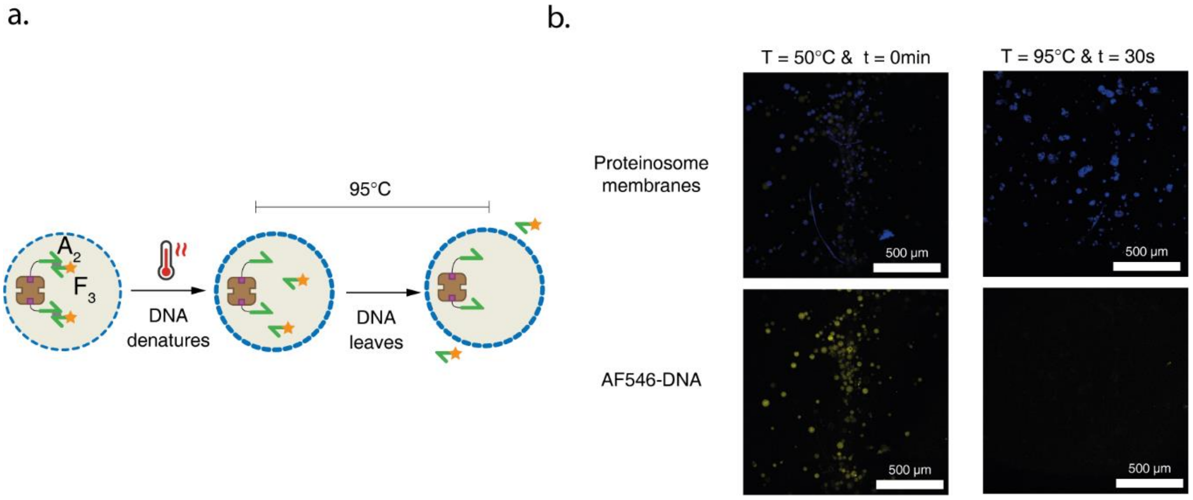

Supplementary Figure 6 Diffusion of 31mer ssDNA over proteinosome membranes is not affected by temperature. a. Cartoon depicting experimental setup. A double-stranded DNA complex (melting temperature 65°C) consisting of 21nt biotin-labelled DNA strand (A2) and 31nt Alexa546 labelled strand (***F_3***) was localized inside proteinosomes. Proteinosomes are subsequently heated to 95°C and subsequently imaged using confocal microscopy. **b.** Confocal micrographs t=0 minutes and 50°C prior to heating and t=30 seconds and 95°C. Sequences for ***A_2*** and ***F_3*** are listed in Supplementary Table 1.

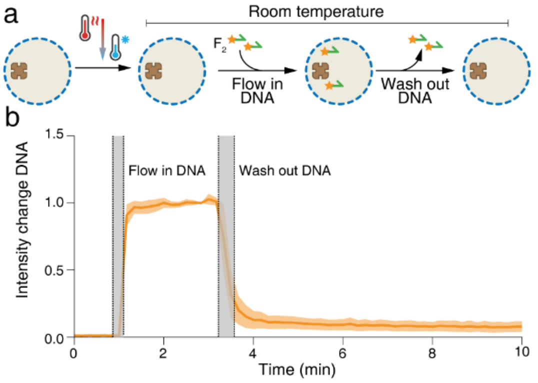

Supplementary Figure 7 Reversibility of membrane collapse. a. Cartoon representation of experiment to determine membrane permeability after heating. Proteinosomes were heated to 95˚C for 10 minutes and subsequently cooled to room temperature before measuring membrane permeability. **b.** Normalized fluorescence intensity plot of DNA inside heated and cooled proteinosomes at room temperature over time (*n=5*). Proteinosomes were heated to 95°C for 10 minutes before cooling to room temperature and conducting the experiment. Proteinosomes were trapped in a microfluidic trapping array[1] and 50 nucleotide long Alexa546-labelled DNA $\boldsymbol{F_2}$ was added. After the fluorescent signal had stabilised, buffer was flowed in to remove excess DNA. Sequence for $\boldsymbol{F_2}$ is listed in Supplementary Table 1.

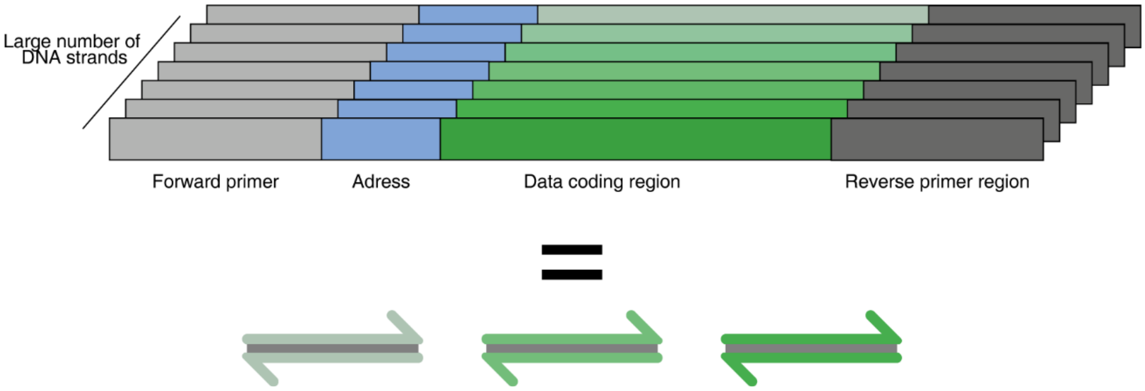

Supplementary Figure 8 Graphical representation of a general DNA file. The procedure for encoding a digital file starts by splitting the original data stream into appropriately sized segments. These segments are then appended with their position within the file such that the segments can be placed in the correct order after sequencing and decoding. To correct the errors inherent to DNA synthesis and sequencing error correction codes are applied to the segments to form the final segments. The final coding segments are then translated into bases in such a way that prevents repeats of identical bases. To the final DNA sequences forward and reverse primer regions are appended to allow for PCR-based amplification and retrieval. The full collection of these strands containing primer regions, address, and coding regions are considered a DNA file in this work. For a detailed description of individual encoding solutions, we refer the reader to references 3–5.

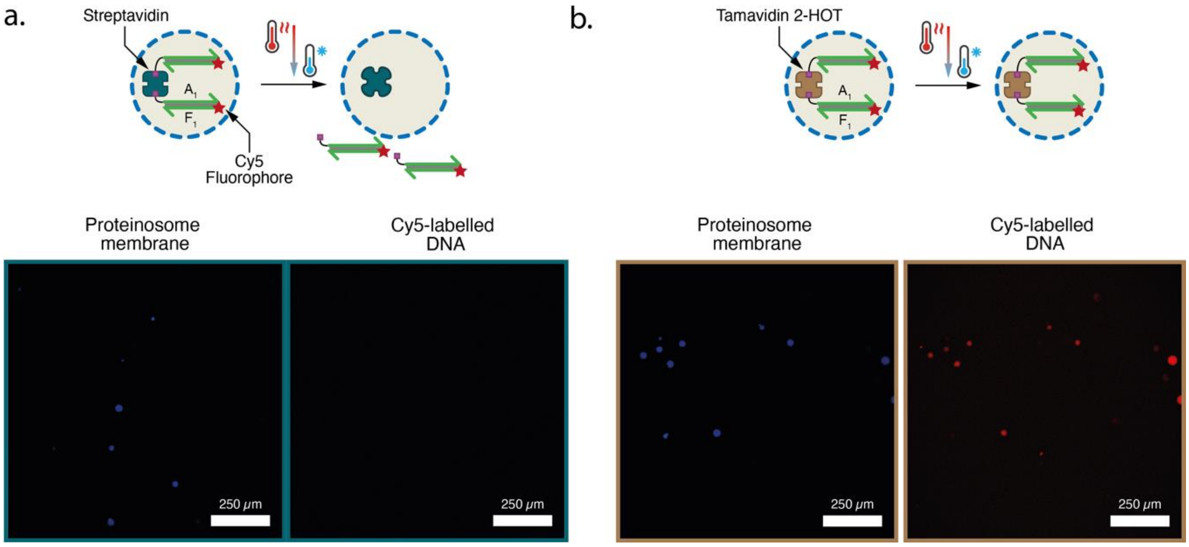

Supplementary Figure 9 Larger versions of micrographs in figure 2a. Confocal micrographs of DNA-containing proteinosomes after heating to 95°C and cooling. Double-stranded DNA (188 base pairs; A1F1) with Cy5 and biotin labels was localized in DyLight405-labelled proteinosomes containing either 4 μM streptavidin (**a.**) or Tamavidin 2-HOT (**b.**), heated to 95°C for 5 minutes, and then cooled to room temperature before imaging. Micrographs of the proteinosome membrane and Cy5-labelled DNA are shown at the left and right of each panel, respectively. Only the proteinosomes containing Tamavidin 2-HOT retained dsDNA. The sequences for strands A1 and F1 are provided in Supplementary Table 1.

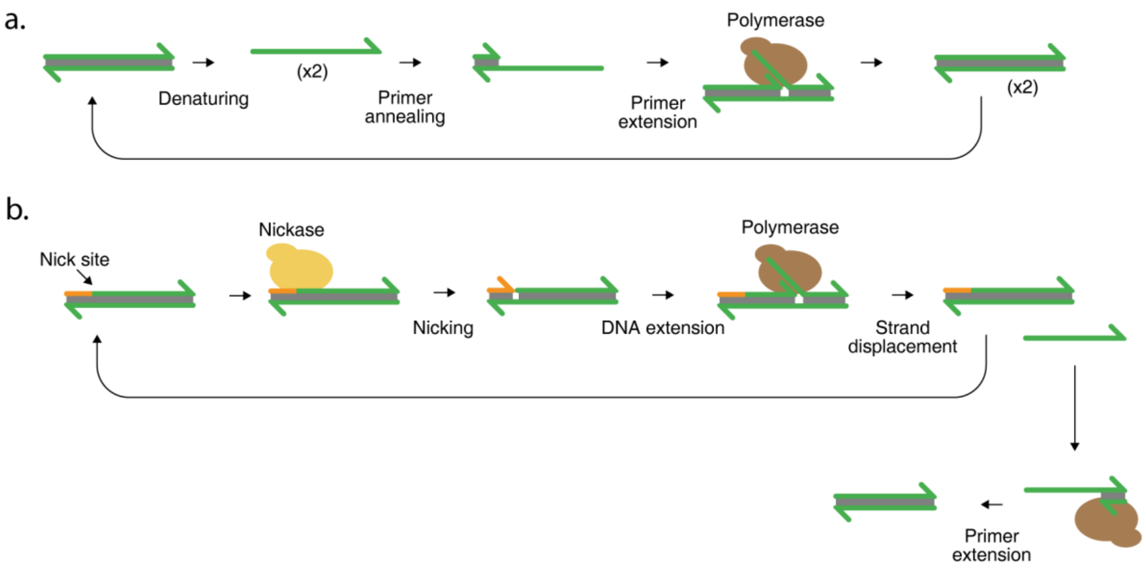

Supplementary Figure 10 Cartoons of DNA amplification methods used. a. Schematic describing polymerase chain reaction (PCR). A piece of dsDNA is first denatured to release two single-stranded DNA molecules (we draw only one of these for clarity). A short DNA primer anneals to the longer template strand and is extended by the DNA polymerase to create a dsDNA copy of the template strand. This process leads to two copies per original dsDNA template and is repeated for a fixed number of cycles. **b.** Schematic describing strand displacement amplification (SDA). A dsDNA template containing a recognition site for a nicking endonuclease (nickase) is cleaved by the nickase to generate a short DNA base-paired to the template strand and a longer secondary template. The primer is extended by a DNA polymerase with high strand displacement activity, thus also displacing the secondary template. The reformed template molecule can then again be nicked and extended to create more secondary template strands. The single-stranded secondary template is converted to dsDNA using an additional primer extension reaction.

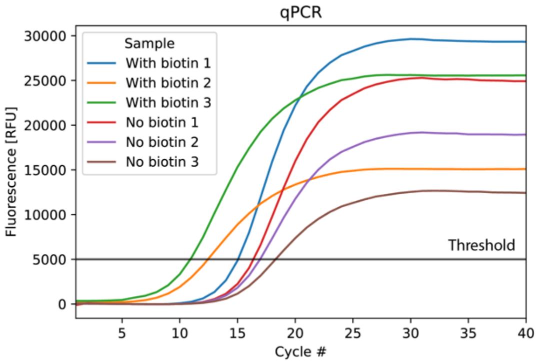

Supplementary Figure 11 Individual traces qPCR experiment summarized in Fig. 3b. Individual traces for all triplicate qPCR experiments of proteinosomes incubated with complexes $\boldsymbol{A_1T_1}$ (with biotin) or $\boldsymbol{U_1T_{1S}}$ (without biotin). Traces were baseline adjusted using the data of the first three cycles. The threshold value was set to 5000 RFU and is plotted as a line to help the reader. Sequences for $\boldsymbol{A_1}$, $\boldsymbol{T_1}$, $\boldsymbol{U_1}$, and $\boldsymbol{T_{1S}}$ are listed in Supplementary Table 2.

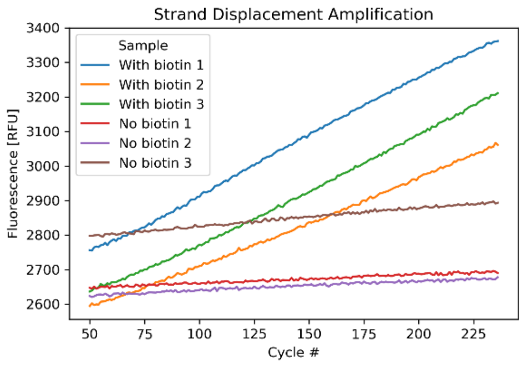

Supplementary Figure 12 Individual traces of strand displacement amplification experiment summarized in Fig. 3c. Fluorescence versus cycle number data for individual SDA reactions of proteinosomes incubated with complexes $\boldsymbol{A_1T_1}$ (with biotin) or $\boldsymbol{U_1T_{1S}}$ (without biotin). Each cycle is approximately 30 seconds since the qPCR machine does not support exporting time-course data, we show cycle numbers. Sequences $\boldsymbol{A_1}$, $\boldsymbol{T_1}$, $\boldsymbol{U_1}$, and $\boldsymbol{T_{1S}}$ are listed in Supplementary Table 2

a.

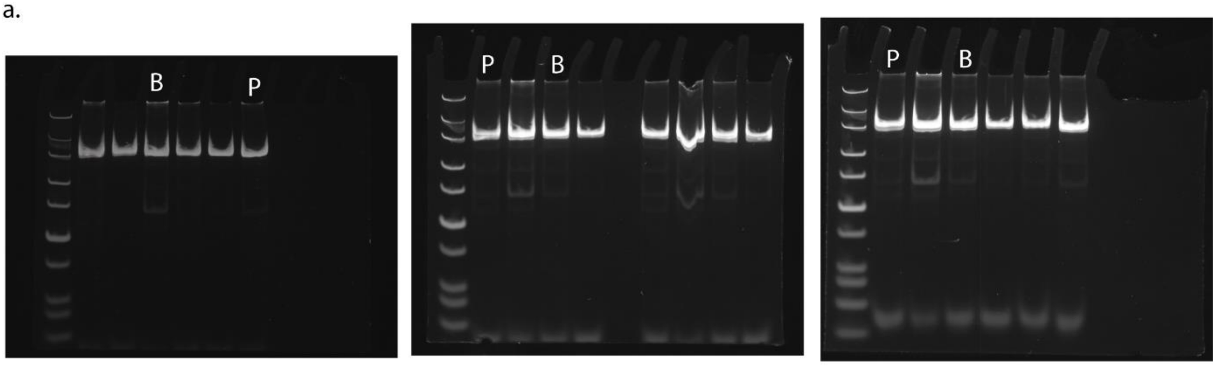

b.

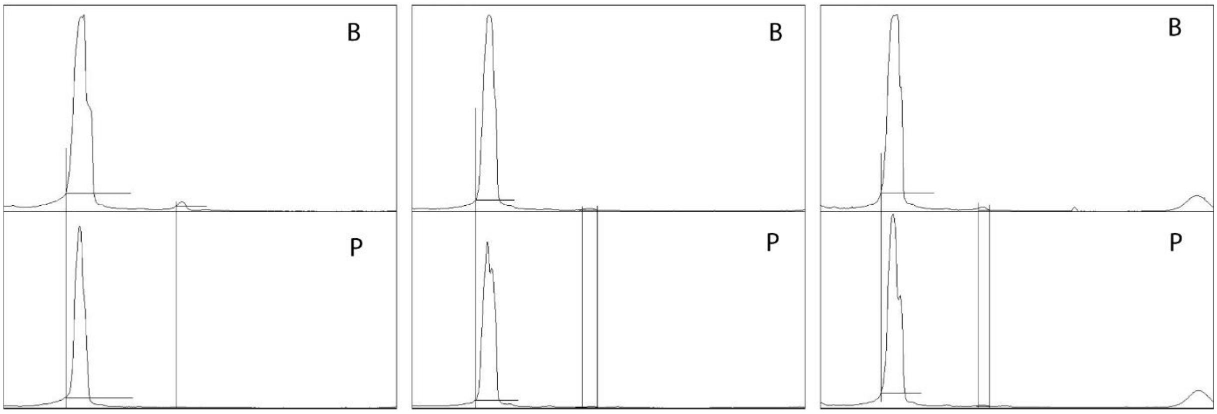

Supplementary Figure 13 Data analysis individual gels Fig. 4d. a. Raw images of independent PAGE gels used to determine ratios of chimaera to target shown in figure 4d. B indicates bulk multiplex PCR, P indicates proteinosome localized amplified DNA. The first gel is the unprocessed image of Fig. 4c, the remaining gels are additional gels run at a later stage. Varying amounts of starting bulk DNA were used such that final concentrations match that of DNA obtained from amplification inside proteinosomes to ensure chimaera formation is not driven solely by differences in concentrations. **b.** FIJI/ImageJ gel analysis plots of lanes

indicated in **a**. Lines were drawn manually to extract the area under the curves for the different products. Ratios of peaks are shown in Fig. 4d.

a. b.

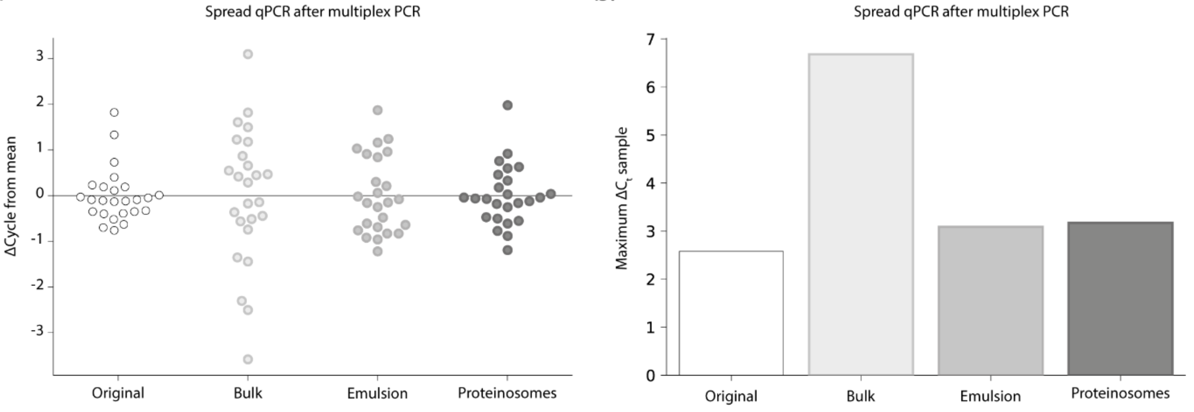

Supplementary Figure 14 PCR inside physically separated compartments maintain the original spread of concentrations in DNA library. a. A library consisting of 25 files encoded in DNA was amplified using either bulk PCR, thermoconfined, or emulsion PCR. These samples were compared against the distribution in the original pool and Ct-values obtained with qPCR were used as an approximation of relative concentrations. **b.** Maximum spread in concentration per condition as determined by qPCR. The lowest Ct-value was subtracted from the highest to represent the maximum fold difference in ΔCt.

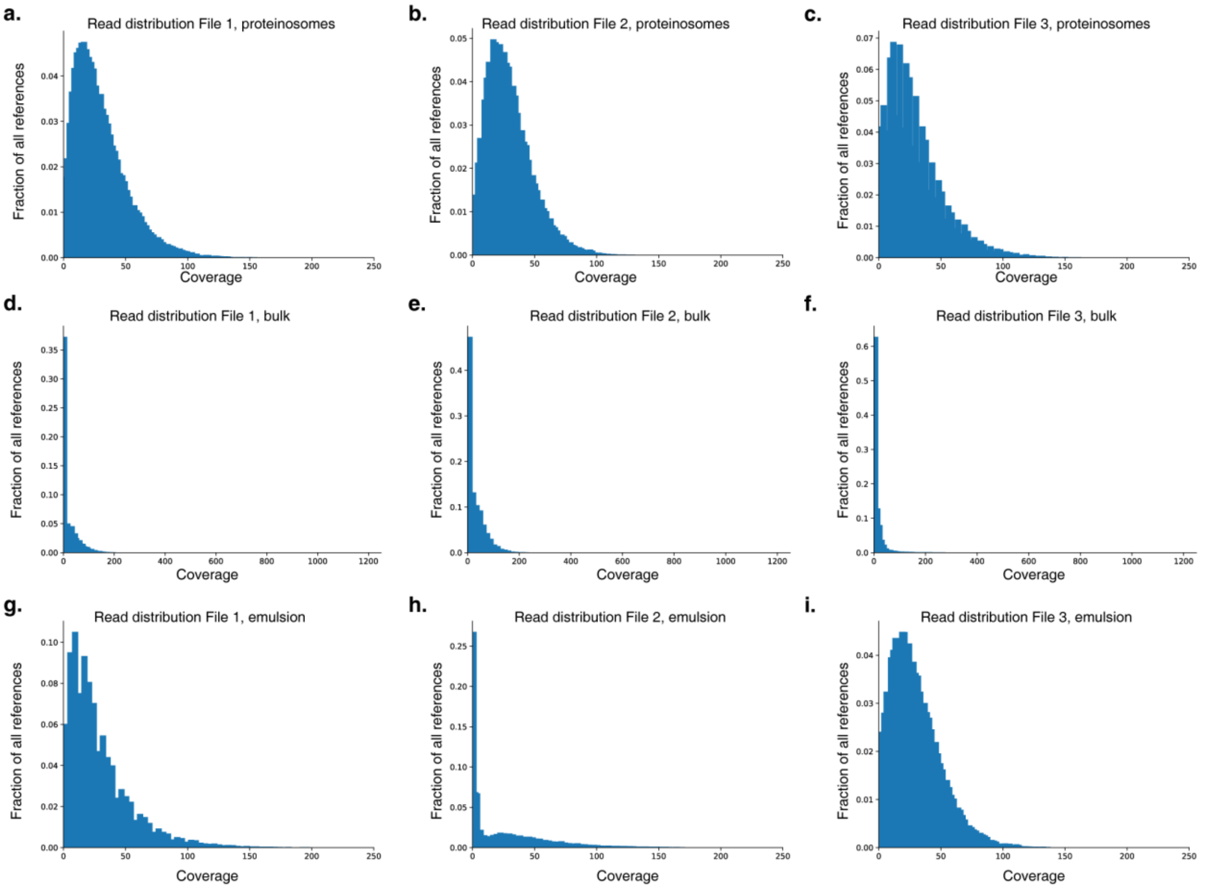

Supplementary Figure 15 Coverage distributions after repeated PCR. Coverages for each unique strand that mapped to a reference sequence used for encoding files are shown as determined by Illumina sequencing, randomly sampled to coverage of 30× for direct comparison. Figures **a.**, **b.** and **c.** show the sequencing coverage distributions for proteinosome-based PCR. Figures **d.**, **e.** and **f.** show the sequencing coverage distributions for bulk amplified DNA **g.**, **h.** and **i.** show the sequencing coverage distributions for emulsion amplified DNA.

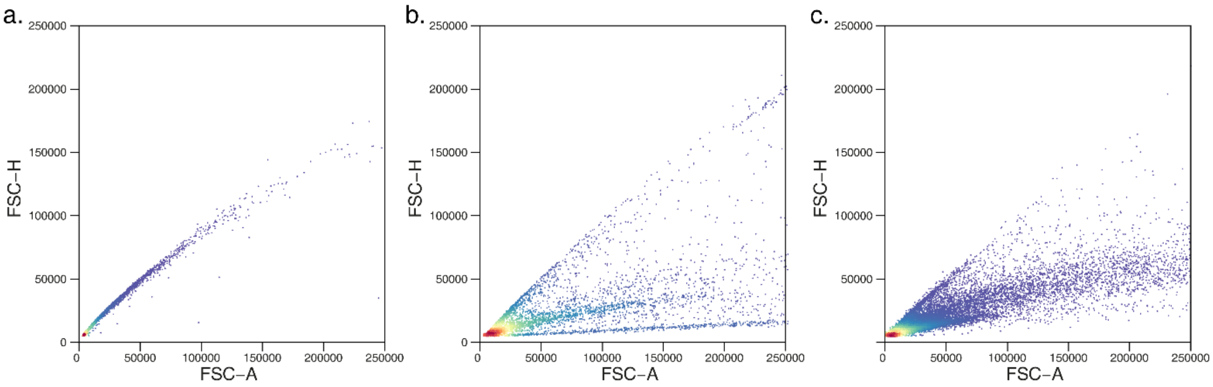

Supplementary Figure 16 Flow cytometry analysis comparing beads and proteinosomes. a. FSC-A versus FSC-H dotplot of magnetic particles used for preparation of proteinosomes (*n=10000*). **b.** FSC-A versus FSC-H dotplot of proteinosomes without magnetic particles (*n=5244*). **c.** FSC-A versus FSC-H dotplot of proteinosomes with magnetic particles (*n=27199*).

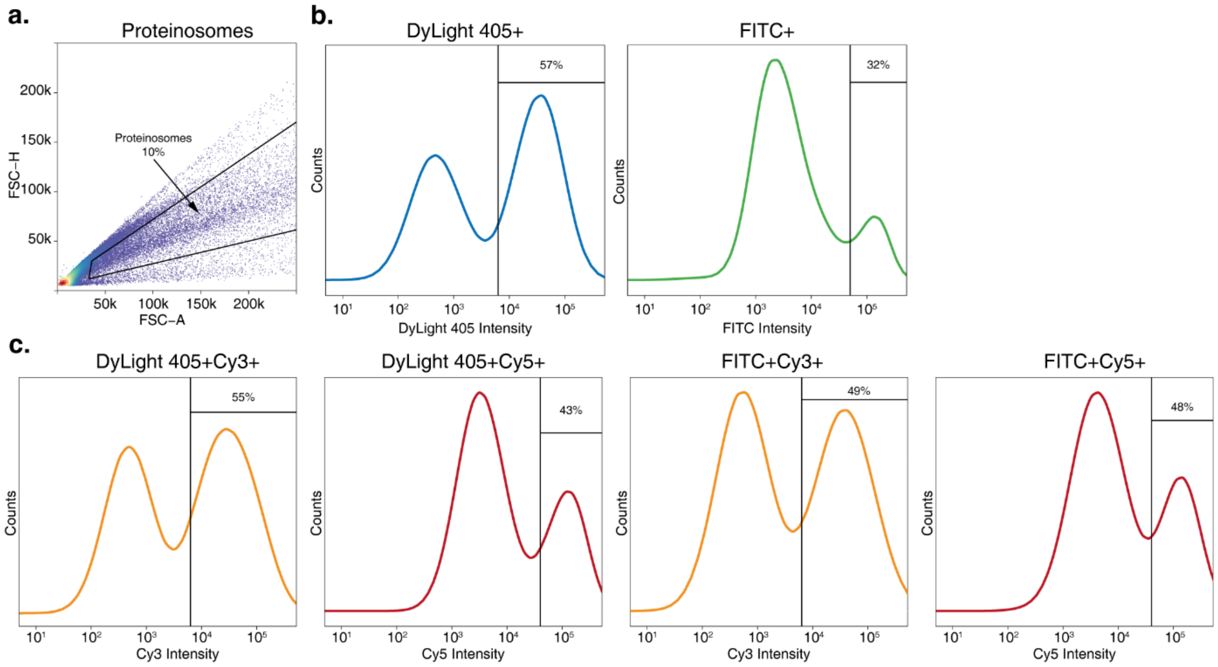

Supplementary Figure 17 Gating strategy used to sort four proteinosome populations. a. FSC-H vs FSC-A dotplot of initially detected events. Based on the experiments shown in Supplementary Fig. 16 a gate was drawn to select proteinosomes against background signal and unincorporated magnetic particles. Proteinosomes accounted for approximately 10% of the total number of detected events. **b.** Histograms of membrane colour intensities detected in proteinosomes gate. Gates were drawn to select high intensity populations in both DyLigh405 and FITC channels. 57% of the total proteinosome population fell within the high DyLigh405 gate, 32% of the total fell within the high FITC gate. **c.** Histograms of DNA barcode fluorescence intensities in high DyLight405 and FITC populations. Proteinosomes gated by membrane colour were subsequently gated in the Cy3 and Cy5 channels to gate based on localized barcodes. In the high DyLight405 populations 55% and 43% of the population was determined to be high Cy3 and high Cy5 respectively. In the high FITC population the high Cy3 population accounted for 49% of the total and high Cy5 for 48%.

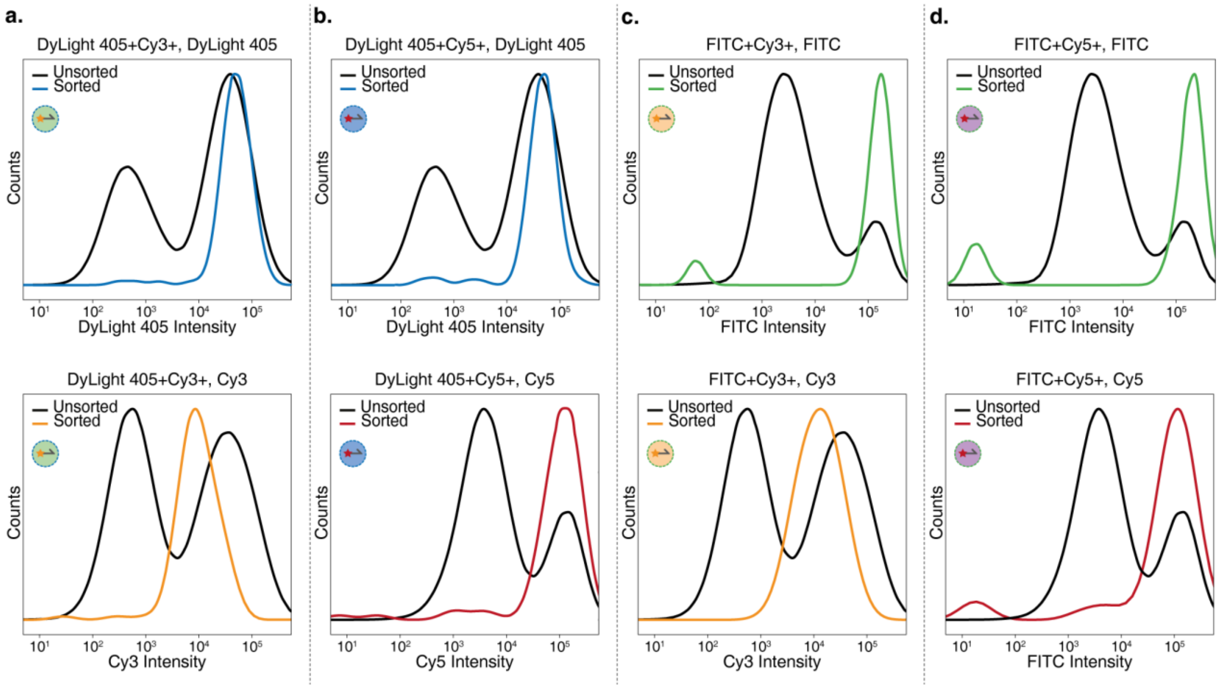

Supplementary Figure 18 Results post-sort flow cytometry. a. DyLight405 (blue) and Cy3 (orange) histograms fluorescence intensities of proteinosomes sorted into high DyLight405 and high Cy3 population (*n=144*). **b.** DyLight405 and Cy5 (red) histograms fluorescence intensities of proteinosomes sorted into high DyLight405 and high Cy5 population (*n=125*). **c.** FITC and Cy3 histograms fluorescence intensities of proteinosomes sorted into high FITC and high Cy3 population (*n=70*). **d.** FITC (green) and Cy5 histograms fluorescence intensities of proteinosomes sorted into high FITC and high Cy5 population (*n=81*). Single peaks are observed after sorting as opposed to the initial bimodal distributions indicating successful sorting for all four populations.

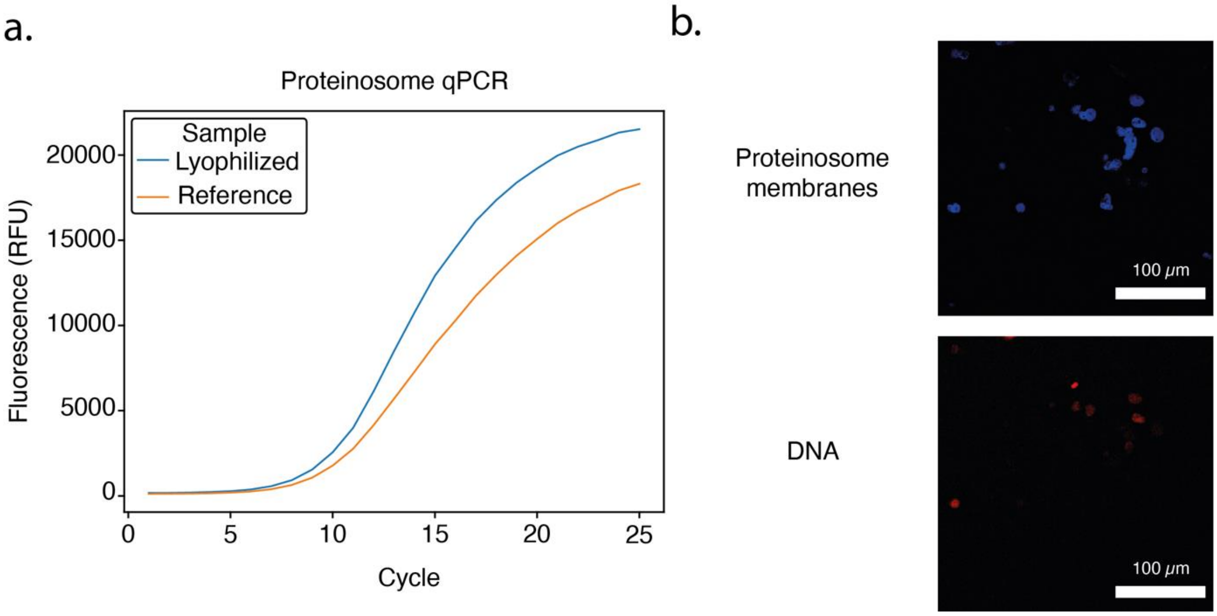

Supplementary Figure 19 Assessing proteinosome stability during lyophilization. a. qPCR plot of proteinosomes containing 4 µM Tamavidin 2-HOT incubated with $\boldsymbol{A_1F_1}$ and subsequently washed five times. One sample was lyophilized and subsequently rehydrated while the control sample was stored at 4˚C. Both show effective DNA amplification, indicating DNA integrity is maintained. Sequences for $\boldsymbol{A_1}$ and $\boldsymbol{F_1}$ are listed in Supplementary Table 1. **b.** Confocal micrographs of lyophilized and subsequently rehydrated proteinosomes. Proteinosome membranes are shown in blue and localized DNA is visualized in red.

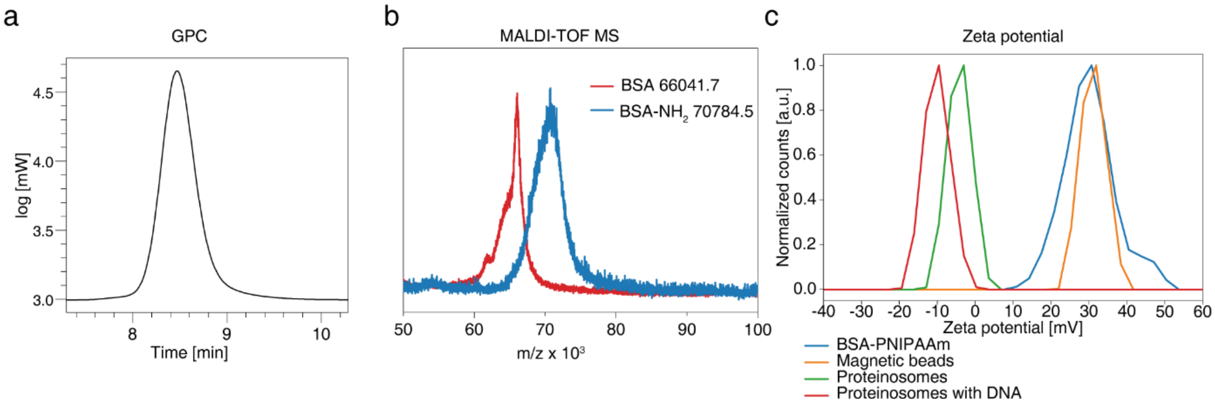

Supplementary Figure 20 Characterization proteinosome components. a. Gel permeation chromatograph of PNIPAAm used to prepare BSA-PNIPAAm conjugates. The molecular weight (M_w) was measured to be 13,284 Da, dispersity was determined to be 1.08. **b.** Matrix-assisted Laser Desorption/Ionization Time of Flight Mass Spectrometry (MALDI-TOF MS) characterization of BSA and cationized BSA. Native BSA had a molecular weight of 66,041.7 Da, after cationization the weight increased to 70,784.5 Da. **c.** Zeta potential measurements of BSA-PNIPAAm, magnetic beads, proteinosomes and DNA-loaded proteinosomes. The BSA-NH_2/PNIPAAm nanoconjugates have a zeta potential of 24.5 mV (Blue). Amine-modified magnetic beads have a zeta potential of 32.1 mV (orange). Empty proteinosomes have a negative zeta potential (-4.45 mV, green) because the BSA-NH_2 molecules in the proteinosome membrane were cross-linked resulting in a reduction in the number of surface amine groups. The charge implies that DNA does not electrostatically bind to the membrane and diffuses

passively into the lumen. The zeta potential of proteinosomes further decreased after localization of negatively charged DNA (-10.5 mV, red).

a.

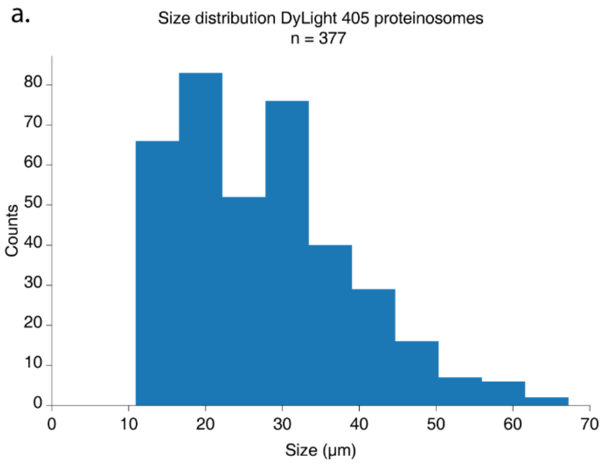

b.

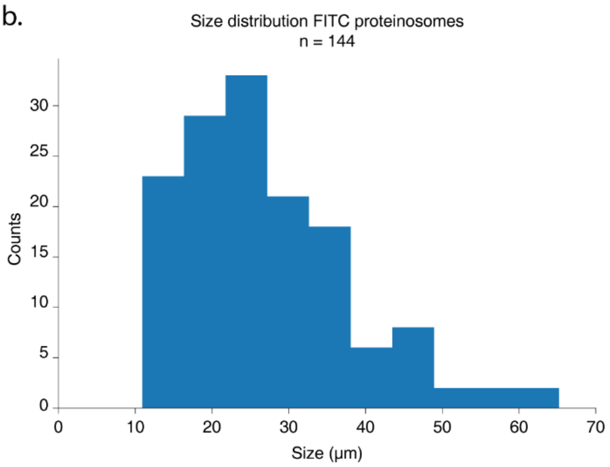

Supplementary Figure 21 Size distribution proteinosomes used for FACS after filtering. a. Size distribution of DyLight 405-labelled proteinosomes. The median diameter was determined to be 25.9 μm. **b.** Size distribution of FITC-labelled proteinosomes. The median diameter was determined to be 25.0 μm.

Supplementary Notes

Supplementary Note 1 Effect dropout on coding density

In our encoding scheme, the outer redundance depends on both the dropout rate and the average rate of errors. Successful decoding is possible if the "error resilience ratio" is at most 1 (please see 5 for details about encoding and decoding). The average number of errors in current sequencing is very small, and here we make such an assumption to simplify the analysis. Then outer redundancy is just slightly higher than the dropout rate. Therefore, when the dropout rate changes from 36.1% to 0.91%; density increases by a factor of (100 – 0.91) / (100-36.1) = 1.56x.

Supplementary Note 2 Estimation of price per MB

For an initial estimate of the added cost per megabyte of our proteinosome-based system. This estimate is based on unit prices of research chemicals, which are not the same as those of similar products at scale. We have also not included the cost of equipment since no speciality devices are required to produce proteinosomes and their per MB price is dependent on the number of proteinosomes produced during the device lifetime, of which we have no estimates. We provide in the table below the cost of chemicals per sales unit and base the cost per proteinosome and MB on this number for the case where one proteinosome is used to encode 1 MB of data, which amounts to hundreds of copies of the sequence used during data encoding. The calculated price is for a 500 µL batch of proteinosomes, which contains approximately 10.000 proteinosomes, leading to a calculated price per MB of stored data of 0.03 Eurocents. This is less than 1% of the cost to synthesize the DNA required to encode 1 MB of data[6].

Component	Unit size	Unit price	Per batch	Price/batch	Reference of price
BSA	100 g	€597.-	0.15 mg	€ 0.001	https://www.sigmaaldrich.com/NL/en/product/sigma/a7030
PNIPAm	5 g	€600.-	0.15 mg	€0.02	https://www.sigmaaldrich.com/NL/en/product/aldrich/725668
Tamavidin 2-HOT	5 mg	€805.-	0.007 mg	€0.08	Discontinued, ordered in 2019
DynaBeads	2 mL	€482.-	2.25 µL	€0.54	https://www.thermofisher.com/order/catalog/product/14307D
2-ethyl hexanol	2.5 L	€65.-	500 µL	€0.02	https://www.sigmaaldrich.com/NL/en/product/mm/800990
Ethanol	2.5 L	€52.90	50 mL	€1.06	https://www.sigmaaldrich.com/NL/en/product/mm/100986
Molecular biology grade water	1 L	€20.-	50 mL	€0.40	https://eu.idtdna.com/pages/products/reagents-and-kits/buffers-and-solutions
Bis-NHS PEG 2K	500 mg	€179.-	1.2 mg	€0.43	https://www.sigmaaldrich.com/NL/en/product/aldrich/713783
Dialysis tubing	30 m	€43.-	3 cm	€0.04	Received quote
			Total	€2.59	

Supplementary Note 3 Estimation of storage density and latency

Since density of data storage is one key argument for DNA storage technology, we here estimated the volumetric density of data stored in proteinosomes and compare it to previous encapsulation methods. The average diameter of a proteinosome is 57 µm (Supplementary Figure 19), which gives a total volume of 97 pL. Since DNA is retained at concentrations of 93.8 nM (Supplementary Figure 2), the total number of DNA molecules inside a proteinosome is

roughly 55.000.000. The average number of DNA strands per 1 MB file used in this work is 65055 strands, so on average proteinosomes can store 840 copies of a single file, which should in principle be enough for perfect decoding after PCR. This equates to a coding density of 8.6 TB/mm^3. Previous work[7] in which DNA was encapsulated in silica glass has shown similar physical densities in the 10 TB/mm^3 range, which while below the theoretical maximal density for a DNA-based data storage system are still higher than those for current technologies.

Supplementary Note 4 Complexity comparison DNA-file compartmentalization methods

We below provide an expanded comparison between PCR-based random access of DNA files using proteinosomes and emulsion PCR. For practical applications, we anticipate proteinosomes are available as off-the-shelf materials.

For the localization of DNA files and PCR using proteinosomes, the protocol is as follows:

1. Localize biotinylated DNA inside proteinosomes (overnight)
2. Addition of first wash buffer and equilibration (overnight)
3. Additional washing using magnetic separator (30 minutes)
4. PCR as normal (20 minutes)
5. Magnetic separation to separate original data and amplicons (3 minutes)

Steps 4 and 5 can, in principle, be repeated as many times as desired and do not require any additional materials than those for regular PCR reactions.

For emulsion PCR, as used in this work, the protocol is as follows:

1. Prepare PCR mixture (supplemented with BSA) and oil mixture (5 minutes)
2. Combine PCR and oil mixtures and vortex to create emulsion (5 minutes)
3. Split emulsion mixture over multiple tubes and run PCR reaction (25 minutes)
4. Pooling of PCR reactions mixtures
5. Breaking emulsion and spin column purification (15 minutes)

All these steps are repeated each time files are accessed.

Additionally, there are two problems that arise during repeated random-access of DNA-encoded files using emulsion PCR.

First, during random access using emulsion PCR, the original DNA files will become mixed with the PCR-generated amplicons. For practical applications, where not all the original DNA files will be amplified, this leads to a situation in which the amplicon pool is not fully representative of the original data-encoded files. Because the original pool is physically sampled for each reaction this in turn leads to a gradual loss of non-amplified, DNA-encoded files from the original pool. In contrast, since after thermoconfined PCR the original data pool can be easily retrieved, file amplification does not lead to pool depletion.

Secondly, the low concentration needed to ensure each emulsion droplet contains a single template molecule complicates repeated random-access using emulsion PCR. In a typical emulsion PCR reaction, the denaturing, annealing, and extension steps are repeated 18 times to ensure sufficient template amplification. After one reaction and subsequent dilution of the

amplicons for a second reaction the fraction of non-amplified files is therefore only $4x10^{-6}$, after two rounds of random-access PCR and dilution this fraction is reduced to $1x10^{-11}$. Combined, the low concentrations and very low abundance of template molecules lead to a situation where sufficient amplification of the target file is extremely challenging. In contrast, since the original data pool can be easily retrieved after thermoconfined PCR, the fraction of non-amplified files is constant in each PCR reaction.

Supplementary Tables

Supplementary Table 1 – DNA sequences used in Figure 2

Name	Sequence	Length (# bases)	5' modification
A1	TTTTTTTTTTTGTCTCACTAGGTCCTCTCAGCTCTTCC CACCACCATTGCCTTCTTCTTCCTTCTGAGTCTAACCA CCACCATTGCCTTATCTACCATTCTGAGTCTTACCAC CACCATTGCCATTTCATTATCTACGAGTCTATCCACC ACCATTGCAGGAGTTTCAGTTTCGACGCAAAAAAAA AA	188	Biotin
F1	TTTTTTTTTTGCGTCGAAACTGAAACTCCTGCAATGG TGGTGGATAGACTCGTAGATAATGAAATGGCAATG GTGGTGGTAAGACTCAGAATGGTAGATAAGGCAAT GGTGGTGGTTAGACTCAGAAGGAAGAAGAAGGCA ATGGTGGTGGGAAGAGCTGAGAGGACCTAGTGAG ACAAAAAAAAAAA	188	Cy5
A2	TGAGAGGACCTAGTGAGACAA	21	Biotin
F2	TAGGTCCTCTCAGCTCTTCCCACCACCATTGCCTTCT TCTTCCTTCTGAG	50	Alexa546
F3	TTTTTTTTTTTTGTCTCACTAGGTCCTCTCA	31	Alexa546

Supplementary Table 2 – DNA sequences used in Figure 3

Name	Sequence	Length (# bases)	5' modification
A1	TTTTTTTTTTTGTCTCACTAGGTCCTCTCAGCTCTTCC CACCACCATTGCCTTCTTCTTCCTTCTGAGTCTAACCA CCACCATTGCCTTATCTACCATTCTGAGTCTTACCAC CACCATTGCCATTTCATTATCTACGAGTCTATCCACC ACCATTGCAGGAGTTTCAGTTTCGACGC	178	Biotin
T1	GCGTCGAAACTGAAACTCCTGCAATGGTGGTGGAT AGACTCGTAGATAATGAAATGGCAATGGTGGTGGT AAGACTCAGAATGGTAGATAAGGCAATGGTGGTGG TTAGACTCAGAAGGAAGAAGAAGGCAATGGTGGTG GGAAGAGCTGAGAGGACCTAGTGAGACAAAAAAA AAAA	178	
U1	TGTCTCACTAGGTCCTCTCAGCTCTTCCCACCACCAT TGCCTTCTTCTTCCTTCTGAGTCTAACCACCACCATT GCCTTATCTACCATTCTGAGTCTTACCACCACCATTG CCATTTCATTATCTACGAGTCTATCCACCACCATTGC AGGAGTTTCAGTTTCGACGC	168	

T1S	GCGTCGAAACTGAAACTCCTGCAATGGTGGTGGAT AGACTCGTAGATAATGAAATGGCAATGGTGGTGGT AAGACTCAGAATGGTAGATAAGGCAATGGTGGTGG TTAGACTCAGAAGGAAGAAGAAGGCAATGGTGGTG GGAAGAGCTGAGAGGACCTAGTGAGACA	168	
Forward primer	TGTCTCACTAGGTCCTCTCA	20	
Reverse primer	GCGTCGAAACTGAAACTCCT	20	

Supplementary Table 3 – DNA sequences used in Figure 4

**Name**	**Sequence**	**Length (# bases)**	**5' modification**
A1	TTTTTTTTTTGCGTCGAAACTGAAACTCCTGCAATGG TGGTGGATAGACTCGTAGATAATGAAATGGCAATG GTGGTGGTAAGACTCAGAATGGTAGATAAGGCAAT GGTGGTGGTTAGACTCAGAAGGAAGAAGAAGGCA ATGGTGGTGGGAAGAGCTGAGAGGACCTAGTGAG ACA	178	Biotin
T1	TGTCTCACTAGGTCCTCTCAGCTCTTCCCACCACCAT TGCCTTCTTCTTCCTTCTGAGTCTAACCACCACCATT GCCTTATCTACCATTCTGAGTCTTACCACCACCATTG CCATTTCATTATCTACGAGTCTATCCACCACCATTGC AGGAGTTTCAGTTTCGACGCAAAAAAAAAA	178	
A2	TTTTTTTTTTGCGTCGAAACTGAAACTCCTACCAATG GGGAGCCGAGGTCATCGAGTGATTACCAGCGGCTA GACTATCTAACGAGCCATTAGAGGCTGACCAGAGC AGACGTACCGCCTGAAATATGCAATGGTGGTGGAT AGACTCGTAGATAATGTGAGAGGACCTAGTGAGAC A	178	Biotin
T2	TGTCTCACTAGGTCCTCTCACATTATCTACGAGTCTA TCCACCACCATTGCATATTTCAGGCGGTACGTCTGCT CTGGTCAGCCTCTAATGGCTCGTTAGATAGTCTAGC CGCTGGTAATCACTCGATGACCTCGGCTCCCCATTG GTAGGAGTTTCAGTTTCGACGCAAAAAAAAAA	178	
C1	TGTCTCACTAGGTCCTCTCACATTATCTACGAGT CTATCCACCACCATTGCAGGAGTTTCAGTTTCG ACGC	71	
C2	GCGTCGAAACTGAAACTCCTGCAATGGTGGTG GATAGACTCGTAGATAATGTGAGAGGACCTAG TGAGACA	71	
Forward primer	TGTCTCACTAGGTCCTCTCA	20	
Reverse primer	GCGTCGAAACTGAAACTCCT	20	

Supplementary Table 4 – DNA sequences used in Figure 5

Name	Sequence	Length (# bases)	5' modification
File 1 Forward	AAAGCGTCGGAAGTTTGTGA	20	
File 1 Reverse	TTTCGCGGTGGTTGTTATGT	20	
File 2 Forward	AACTGCTTAATCGTGCCACA	20	
File 2 Reverse	ACAAACTTCGAGAACTGCGT	20	
File 3 Forward	ATGAGTGCGTCTTGTGTGTT	20	
File 3 Reverse	ACCGCTTTGACGCAAACTAA	20	
File 4 Forward	TTAATCGGTAACACCTGCGG	20	
File 4 Reverse	CACTTTAGCCTAACGGTGGT	20	
File 5 Forward	ACTGCCAACGTATTGCACAT	20	
File 5 Reverse	AAACCAGACCGTTGTCGAAA	20	
File 6 Forward	AATTTGGCATTCACCGTGGA	20	
File 6 Reverse	TTTCATTGGCTTGCACCAGA	20	
File 7 Forward	TCACGACTTTGTTTGCGAGT	20	
File 7 Reverse	AAGTTAGTTCAACGTCGCGT	20	
File 8 Forward	TTGGTTGACCGTGTGTGAAT	20	
File 8 Reverse	ATAACGCGCAAGAAGCATCT	20	
File 9 Forward	AATGAAGATGCACGCCTTGA	20	
File 9 Reverse	ACAAGCATTCCGTCACAGTT	20	
File 10 Forward	AATGGACGTTCCGCAATCAT	20	
File 10 Reverse	ATTAGCCAAACCATAGCGCA	20	
File 11 Forward	TTACCGTGCAGTTGACGAAA	20	
File 11 Reverse	ATTGTTACGAATCGGTGCCA	20	
File 12 Forward	ATCTGCGCTTAACAAGGCTT	20	
File 12 Reverse	AGTCGCCAAATAAGTGCCAT	20	
File 13 Forward	TGGTTTCGTGTTCAAGCGTA	20	
File 13 Reverse	CGCCATTGCAGAAAGAGAGA	20	
File 14 Forward	AAACAAAGTTAGCGGCTCGT	20	
File 14 Reverse	AATAACCGCGAATTGGCCTT	20	
File 15 Forward	TGGTTTGCAGGAAATGTCGT	20	
File 15 Reverse	ACCGCTTTGCACTCAGTTAA	20	
File 16 Forward	ACCGCGCTCGAAGAATTTAA	20	
File 16 Reverse	TACAGGCAACAAACGCATCT	20	
File 17 Forward	TCTCCGGTGCTACGTTAAAG	20	
File 17 Reverse	CGGCCTTCTCCAAAGAATCA	20	
File 18 Forward	AACGAATCGAACTGCCTTGT	20	
File 18 Reverse	TGAAACATATTCGCACGCCT	20	
File 19 Forward	TCCTGCTTGCGTTAAATGGA	20	
File 19 Reverse	AAACCGACGGAAACATTGCA	20	
File 20 Forward	AACCGCTTTGGCTCAGTATT	20	
File 20 Reverse	TAAAGGCAGGCAAACGGATT	20	
File 21 Forward	AGTGCATTCTCCAAGCAACT	20	

File 21 Reverse	TACACACGGTTTGGCTTGAA	20	
File 22 Forward	TTCGCAGGTGACTTTGTGTT	20	
File 22 Reverse	ATGAGGCAAATCGTCGCAAA	20	
File 23 Forward	AACCGATGCGCTTATACGTT	20	
File 23 Reverse	AGGAAGCGCCAACTAATTGT	20	
File 24 Forward	AGCCTTGTGTCCATCAATCC	20	
File 24 Reverse	TTGTTCAGTGGAAGACGACG	20	
File 25 Forward	AAGGCTCGCGTGCATATTAA	20	
File 25 Reverse	ATTATGAAAGCGCAGGCAGA	20	
File 1 Forward bio	TTTTTTTTTTTAAAGCGTCGGAAGTTTGTGA	31	Biotin
File 2 Forward bio	TTTTTTTTTTTAACTGCTTAATCGTGCCACA	31	Biotin
File 3 Forward bio	TTTTTTTTTTTATGAGTGCGTCTTGTGTGTT	31	Biotin
File 4 Forward bio	TTTTTTTTTTTTTAATCGGTAACACCTGCGG	31	Biotin
File 5 Forward bio	TTTTTTTTTTTACTGCCAACGTATTGCACAT	31	Biotin
File 6 Forward bio	TTTTTTTTTTTAATTTGGCATTCACCGTGGA	31	Biotin
File 7 Forward bio	TTTTTTTTTTTTCACGACTTTGTTTGCGAGT	31	Biotin
File 8 Forward bio	TTTTTTTTTTTTTGGTTGACCGTGTGTGAAT	31	Biotin
File 9 Forward bio	TTTTTTTTTTTAATGAAGATGCACGCCTTGA	31	Biotin
File 10 Forward bio	TTTTTTTTTTTAATGGACGTTCCGCAATCAT	31	Biotin
File 11 Forward bio	TTTTTTTTTTTTTACCGTGCAGTTGACGAAA	31	Biotin
File 12 Forward bio	TTTTTTTTTTTATCTGCGCTTAACAAGGCTT	31	Biotin
File 13 Forward bio	TTTTTTTTTTTTGGTTTCGTGTTCAAGCGTA	31	Biotin
File 14 Forward bio	TTTTTTTTTTTAAACAAAGTTAGCGGCTCGT	31	Biotin
File 15 Forward bio	TTTTTTTTTTTTGGTTTGCAGGAAATGTCGT	31	Biotin
File 16 Forward bio	TTTTTTTTTTTACCGCGCTCGAAGAATTTAA	31	Biotin
File 17 Forward bio	TTTTTTTTTTTTCTCCGGTGCTACGTTAAAG	31	Biotin
File 18 Forward bio	TTTTTTTTTTTAACGAATCGAACTGCCTTGT	31	Biotin
File 19 Forward bio	TTTTTTTTTTTTCCTGCTTGCGTTAAATGGA	31	Biotin
File 20 Forward bio	TTTTTTTTTTTAACCGCTTTGGCTCAGTATT	31	Biotin
File 21 Forward bio	TTTTTTTTTTTAGTGCATTCTCCAAGCAACT	31	Biotin
File 22 Forward bio	TTTTTTTTTTTTTCGCAGGTGACTTTGTGTT	31	Biotin
File 23 Forward bio	TTTTTTTTTTTAACCGATGCGCTTATACGTT	31	Biotin
File 24 Forward bio	TTTTTTTTTTTAGCCTTGTGTCCATCAATCC	31	Biotin
File 25 Forward bio	TTTTTTTTTTTAAGGCTCGCGTGCATATTAA	31	Biotin

Supplementary Table 5 – Coverage per file for each condition of plot in figure 5b

File	Original	Bulk	Emulsion	Proteinosomes
1	16.23	5.50	27.76	8.77
2	20.63	14.94	32.17	13.82
3	20.85	12.19	32.49	12.08
4	23.70	15.04	29.00	31.80
5	26.88	21.53	36.11	18.56
6	17.61	199.91	73.12	13.30
7	16.54	84.48	44.78	12.89
8	9.82	33.29	15.29	5.84
9	30.69	46.80	64.38	38.47
10	21.47	11.21	31.92	21.15
11	25.61	78.26	57.12	26.19
12	24.35	13.04	37.15	17.57
13	15.83	25.65	28.33	12.84
14	18.09	11.20	30.17	9.28
15	24.81	5.22	22.66	26.77
16	18.83	16.58	37.76	11.97
17	15.89	16.81	34.17	5.42
18	26.40	14.19	45.95	20.08
19	15.28	19.03	36.72	8.10
20	19.21	3.31	21.36	18.41
21	17.78	19.98	37.87	9.39
22	22.16	7.15	33.21	6.06
23	23.44	31.91	44.18	25.13
24	16.42	17.45	32.13	15.49
25	27.79	9.58	34.36	17.57

Supplementary Table 6 – Sequences used in figure 6

Name	Sequence	Length (# bases)	5' modification	3' modification
F4	GACAGGAAAGGACTCGGTGGGT TAGGCAATGGATCAG	37	Biotin	Cy3
F5	GTTAGACTCTTAGGGTGGTGC AATGGGGTAT	31	Biotin	Cy5
File 1 Forward	TGGTTTCGTGTTCAAGCGTA	20		
File 1 Reverse	CGCCATTGCAGAAAGAGAGA	20		
File 2 Forward	AAACAAAGTTAGCGGCTCGT	20		
File 2 Reverse	AATAACCGCGAATTGGCCTT	20		
File 3 Forward	TGGTTTGCAGGAAATGTCGT	20		
File 3 Reverse	ACCGCTTTGCACTCAGTTAA	20		
File 4 Forward	ACCGCGCTCGAAGAATTTAA	20		

File 4 Reverse	TACAGGCAACAAACGCATCT	20		

Supplementary references

1. Joesaar, A. *et al.* DNA-based communication in populations of synthetic protocells. *Nature Nanotechnology* (2019) doi:10.1038/s41565-019-0399-9.
2. Yang, S., Joesaar, A., Bögels, B. W. A., Mann, S. & de Greef, T. F. A. Protocellular CRISPR/Cas-Based Diffusive Communication Using Transcriptional RNA Signaling. *Angewandte Chemie International Edition* **61**, e202202436 (2022).
3. Goldman, N. *et al.* Towards practical, high-capacity, low-maintenance information storage in synthesized DNA. *Nature* **494**, 77–80 (2013).
4. Erlich, Y. & Zielinski, D. DNA Fountain enables a robust and efficient storage architecture. *Science* **355**, 950–954 (2017).
5. Organick, L. *et al.* Random access in large-scale DNA data storage. *Nature Biotechnology* **36**, 242–248 (2018).
6. Antkowiak, P. L. *et al.* Low cost DNA data storage using photolithographic synthesis and advanced information reconstruction and error correction. *Nat Commun* **11**, 5345 (2020).
7. Grass, R. N., Heckel, R., Puddu, M., Paunescu, D. & Stark, W. J. Robust Chemical Preservation of Digital Information on DNA in Silica with Error-Correcting Codes. *Angewandte Chemie International Edition* **54**, 2552–2555 (2015).
8. Meiser, L. C. *et al.* Reading and writing digital data in DNA. *Nat Protoc* **15**, 86–101 (2020).

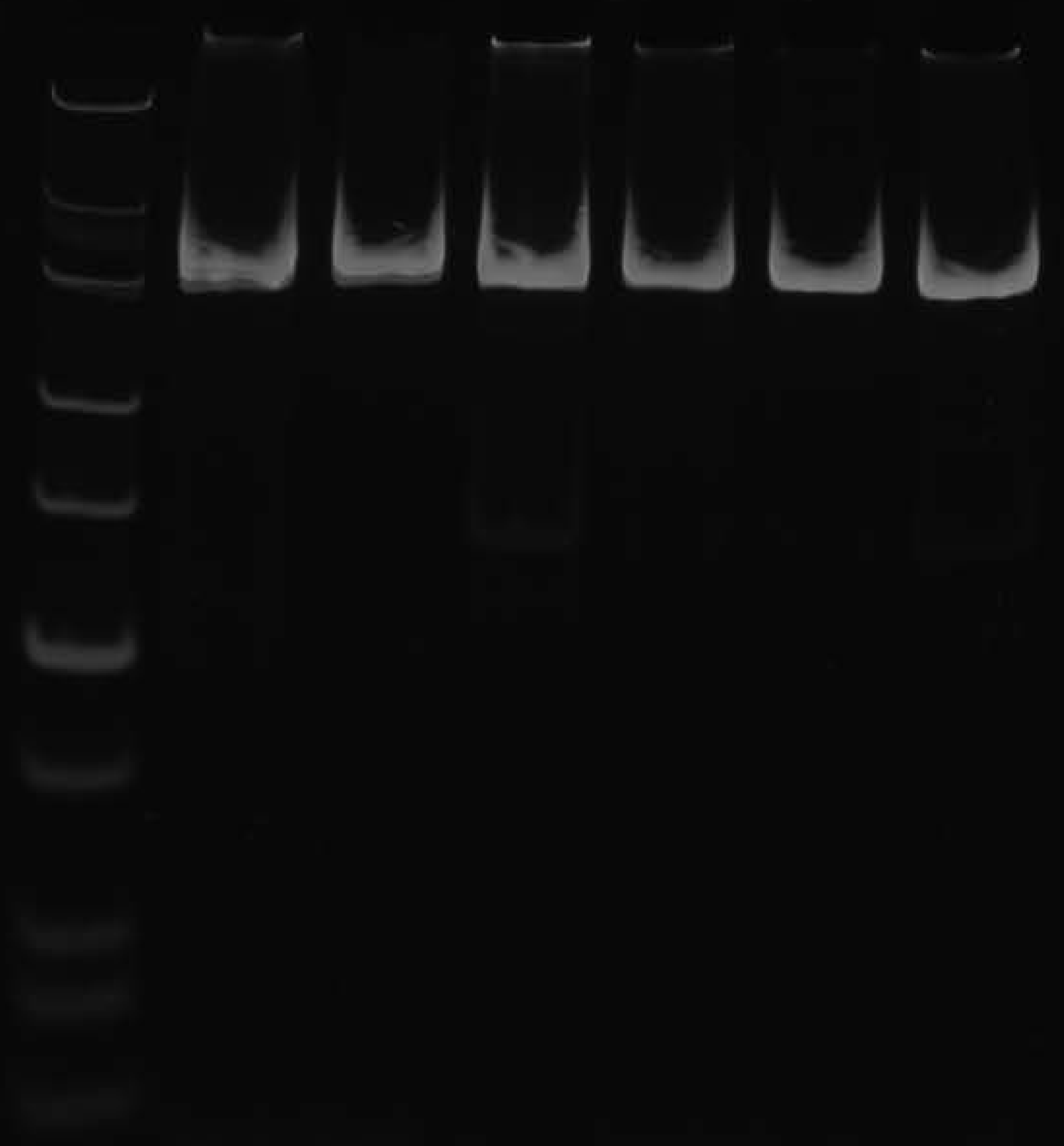

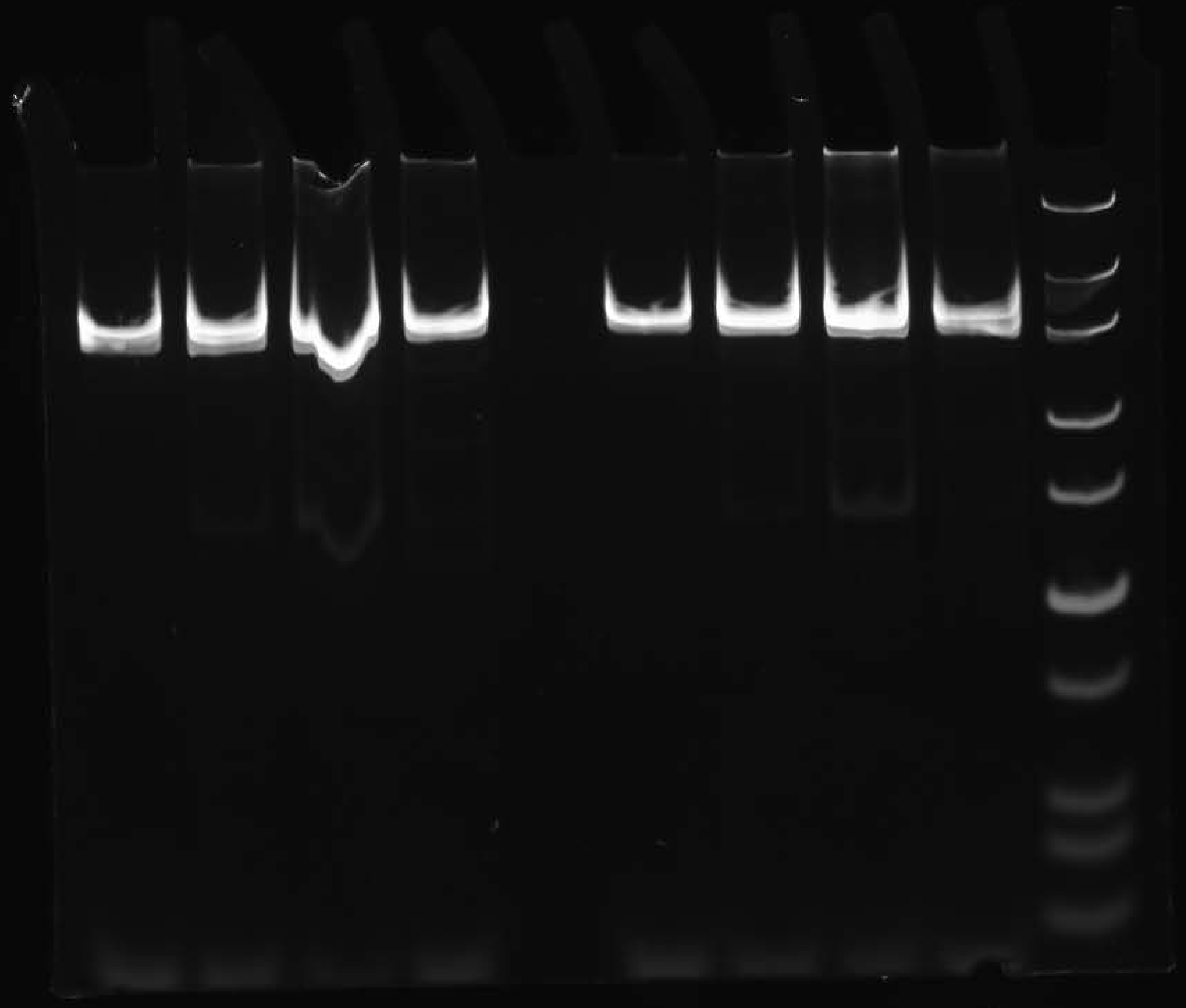

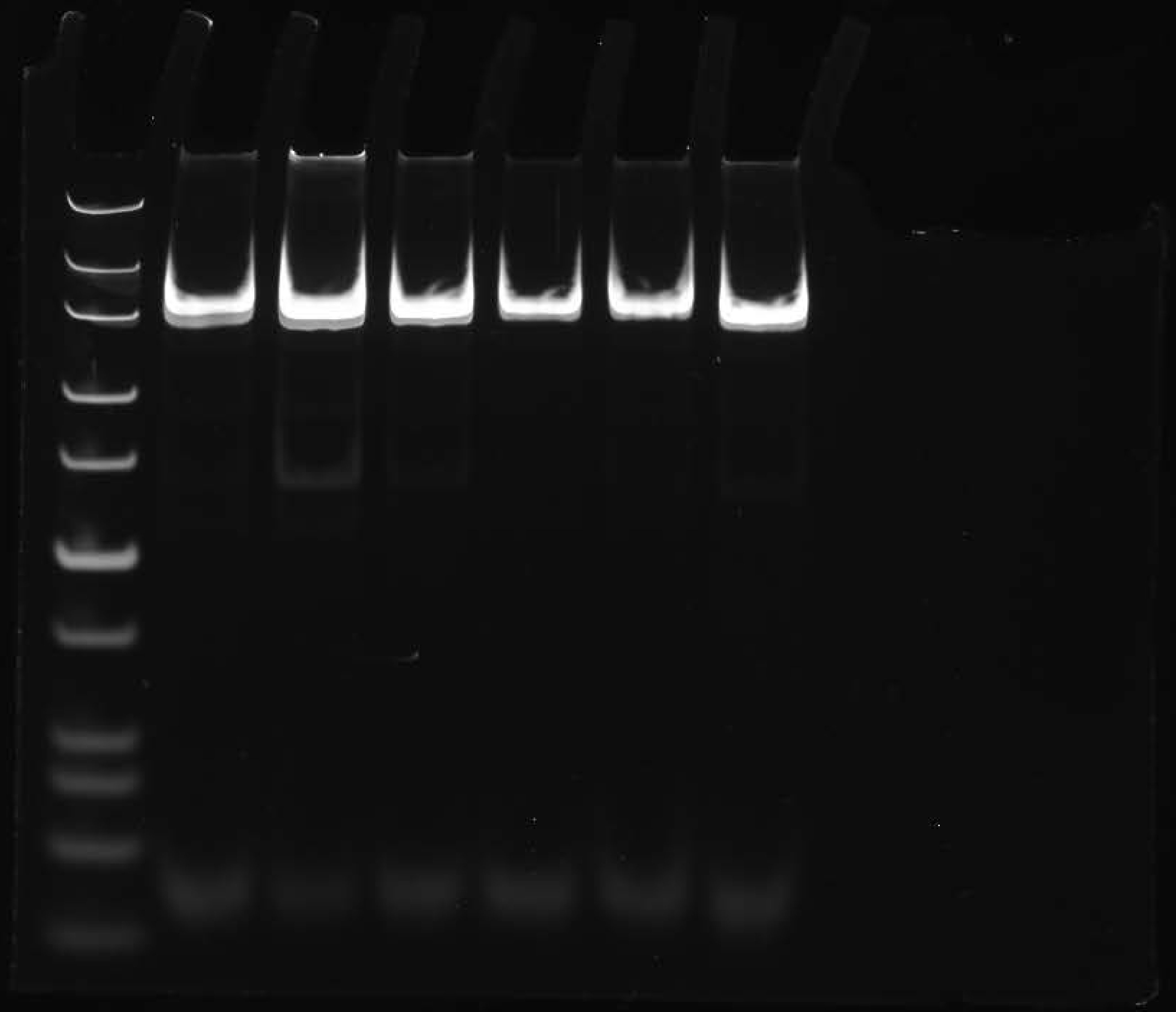

Supplement: Supplementary file 1 — Supplementary Figs. 1–21, Notes 1–4, Tables 1–6, Unprocessed gels 1–4 and methods. [file 41565_2023_1377_MOESM1_ESM.pdf]
