# Supplementary figures and images for: DNA storage in thermoresponsive microcapsules for repeated random multiplexed data access
Source: Nat Nanotechnol. 2023 May 4;18(8):912–21. doi: 10.1038/s41565-023-01377-4 (PMC10427423; doi:10.1038/s41565-023-01377-4)

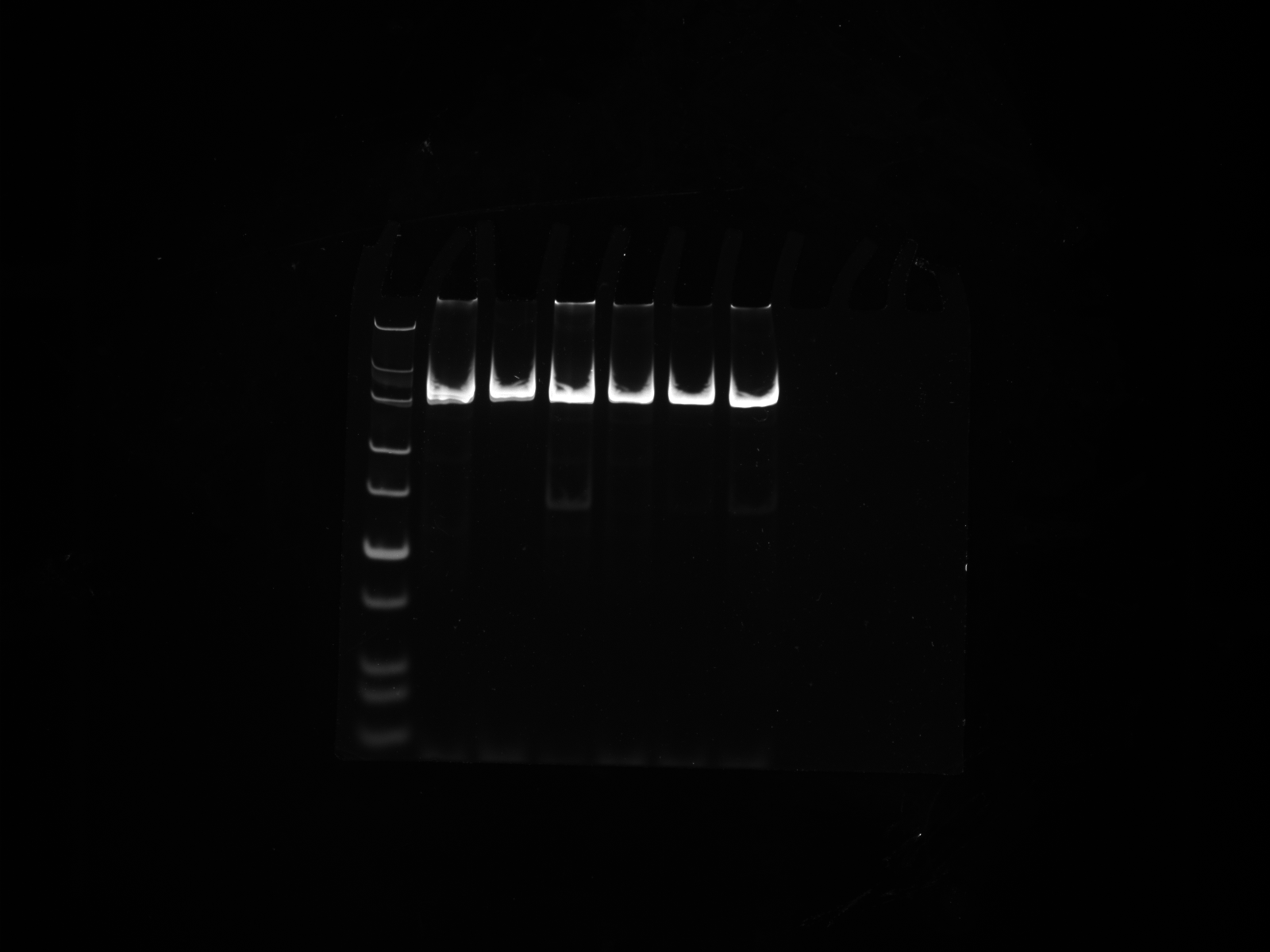

Supplement: Source Data Fig. 4 — Raw data points of all the graphs presented in Fig. 4. Data are ordered with a separate tab for the figure and correspondingly labelled as well as for the unprocessed PAGE gel of the chimeric PCR experiment. The processed version is shown in Fig. 4b. [file 41565_2023_1377_MOESM5_ESM.zip › Source_data_fig4/PAGE_gel_chimera_1.tif]
